# A simulation and modeling approach of coupled thermal and electrical behavior of PV panels using the artificial hummingbird algorithm and two-dimensional finite difference-based model

**DOI:** 10.1016/j.heliyon.2024.e27244

**Published:** 2024-03-04

**Authors:** Radouane Aalloul, Abdellah Elaissaoui, Assia Harkani, Rhma Adhiri, Mourad Benlattar

**Affiliations:** a Laboratory of Engineering and Materials (LIMAT), Faculty of Sciences, Ben M'Sick Hassan II University of Casablanca, Casablanca, 20000, Morocco; b Laboratory of Agricultural Engineering, Energy National Institute of Agricultural Research Settat, Settat, 26000, Morocco; c Matter Physics Laboratory (LPM), Faculty of Sciences, Ben M'Sick Hassan II University of Casablanca, Casablanca, 20000, Morocco

**Keywords:** Photovoltaic, Double-diode model, Finite difference, Electrical-thermal, Parameter extraction

## Abstract

Accurate estimation of photovoltaic (PV) panels’ temperature is crucial for an accurate assessment for both the electrical and thermal aspects and performances. In this study we propose an advanced simulation approach linking a double-diode (DD) electrical model using the Artificial hummingbird algorithm; for parameter extraction; and a two-dimensional finite-difference-based thermal model. The electrical-sub model is firstly validated in comparison to experimental data figuring in literature using three types of PV technologies, with a relative error of about 2%. Then, the coupled model is validated using in-situ experimental setup consisting of the usage of thin-film PV technology, temperature sensors, weather station and an infrared camera. The results from both simulations and experiments exhibit strong alignment with a relative error of not higher than 2%; mainly due to the used material calibration uncertainties and external perturbations. This holistic model can be indeed further optimized, still, it has a potential to advance the development in the research area of PV systems.Future efforts could involve additional experimentation to validate the model for different seasons of the year.

## 1 Introduction

Starting with the second industrial revolution in 1870, electricity has played a major role in our daily lives. This electricity was mainly produced using fossil fuels. However, given the increasing environmental concern and the drastic rise in energy demand, transitioning towards clean energy production is becoming imperative [1]. Among these sources, we can mention photovoltaic (PV) technologies that convert solar energy into electricity. However, their power output usually fluctuates in response to solar irradiation, surface temperature and other weather conditions, making them less favored in terms of ensuring a continuous electricity supply [2]. Hence, it is important to study their performance beforehand and predict how they will respond to real weather conditions. Photovoltaic panel performance is highly susceptible to variable weather conditions, including factors such as cloud cover, shading, temperature fluctuations, and changes in solar irradiance. Moreover, the stock of dust and pollution on panel surfaces can lower their efficiency. In addition to this, PV panels degrade over time due to exposure and environmental influences. Recognizing the rate of degradation and its effect on long-term operation forecast is what mainly presents the challenge. Last but not least, PV technologies continuously evolve, hence, new challenges arise in terms of understanding their behavior concerning designs and the materials used.

### 1.1 State-of-the-art

#### 1.1.1 Electrical modeling

In relevant literature review, different models exist and are used to explain the behavior and performances of PV technologies. In Ref. [3], M. G. Villalva et al. suggested a control approach for grid-connected single-phase PV systems that attempts to increase their power quality and efficiency. The technique calls for the implementation of a feedforward control loop that evaluates the instantaneous power output of the PV array and changes the inverter's current reference correspondingly.

**Table:** 

Nomenclatures			
AHA	Artificial hummingbird algorithm	K	Boltzmann constant
APSO	adaptive particle swarm optimization	MA	Metaheuristic Algorithm
AR-ADE	onlooker-ranking-based adaptive differential evolution	MAPE	a mean absolute percentage error
ASHRAE	American Society of Heating, Refrigerating and Air-Conditioning Engineers	MDM	Multiple-Diode Model
CIGS	copper indium gallium selenide	MPP	maximum power point
CPSO	assured convergence particle swarm optimization	NOCT	nominal operating conditions temperature
CPV	concentrated photovoltaic	PSO	Particle Swarm Optimization
DC	Direct current	PTC	PVUSA test conditions
DD	Double diode	PV	Photovoltaic
DDM	Double-Diode Model	PVT	Polyvinyl fluoride
DE	Differential Evolution	RMSE	root mean square error
FF	fill factor	SDM	Single-Diode Model
GA	Genetic Algorithm	STC	standard test conditions
GPOA	plane-of-array irradiation	TPT	Tedlar Polyester Tedlar
GSTC	solar irradiance under standard conditions	TSTC	temperature under standard conditions
INRA	National Institute for Agricultural Research	UDF	user defined functions
I–V	Current Voltage	VT	thermal voltage of the PV module
		EVA	ethylene vinyl acetate

The electrical model of the PV system used in the study consists of a DC/DC converter, a DC link, and a single-phase inverter, while the electrical model of the PV array is a five-parameter model consisting of a current source, a diode, a series resistance, and a shunt resistance. The suggested technique was shown to be efficient in correcting the harmonic distortion generated by the nonlinear behavior of the inverter and the power factor deviation caused by the variable nature of the PV source. Through comparison between experimental and simulation results, it has been shown that the suggested technique has the potential to improve the performance and dependability of single-phase PV systems linked to the grid, and it may be applied to other renewable energy systems with similar nonlinear and variable characteristics.

In [4], authors examined the impact of temperature and irradiance on the electrical performance of a commercial PV module. Using conventional I–V and P–V curve measurements, it was shown that a rise in temperature reduces the maximum power output of the PV module owing to a drop in open-circuit voltage and an increase in short-circuit current. In contrast, a rise in irradiance increases the maximum power output of the module owing to an increase in both the open-circuit voltage and the short-circuit current. It has also been noticed that the PV module's fill factor falls with rising temperature but rises with increased irradiation. While the research did not use a particular electrical model of a PV system, the results may be utilized to enhance current models and maximize the performance and efficiency of PV systems.

Authors in Ref. [5] discuss PV module modeling methods and emphasize the importance of precision for the system to be efficient. They note that empirical models, which are based on PV module electrical behavior under various environmental circumstances, are most widely employed to forecast PV module performance. However, they are limited in their application to various PV modules and are less accurate under varied climatic circumstances. Authors also compare between analytical and numerical models in terms of complexity, accuracy and time response.

In [6], authors present the most commonly used diode-based models for simulating the electrical properties of PVs, while stressing the need for accuracy to enhance systems performance. According to the research, the I–V properties of a PV cell are often modeled using the Shockley diode equation. Parameters such as the diode's saturation current, ideality factor, and thermal voltage are utilized in the equation to characterize the relationship between the current and voltage across a diode. This work highlights that the Shockley diode equation is simplified when using only one diode, but becomes more complex if two diodes are involved. Lastly, the authors address the shortcomings of diode models, such as their inability to account for spatial changes in the PV cell's electrical characteristics.

In [7], Jordehi offers a complete overview of the various methodologies utilized for solar PV cell parameter assessment. The study examines the lumped, distributed, and empirical electrical models used to describe the behavior of PV cells. The report suggests the adoption of appropriate electrical models depending on application-specific needs and data availability. The author underlines the significance of selecting the proper number of model parameters to prevent overfitting or underfitting of the data. To circumvent the limits of conventional methodologies, the research suggests using sophisticated optimization algorithms (such as genetic algorithms and particle swarm optimization) for parameter estimation in addition to a multi-set of experimental data as to confirm the predicted parameters and strengthen the model's precision.

Ghani et al. in Ref. [8] developed and validated an electrical model of crystalline silicon PV systems using manufacturer-supplied data. To simulate the electrical behavior of PV devices under varying working circumstances, they used the frequently deployed single-diode, double-diode, and comparable circuit models. To verify the accuracy of the models, authors compared the simulated outcomes to the manufacturer's experimental data. In addition, they evaluated the models' sensitivity to fluctuations in key electrical characteristics, such as the ideality factor, series resistance, and shunt resistance. This analysis helped them identify the most crucial parameters for accurate modeling.

Using a multi-objective adaptive genetic algorithm to estimate the unidentified parameters of a single-equivalent diode's circuit model, Kumari and Geethanjali established an optimization framework for PV cell parameter extraction in Ref. [9]. The goal of the optimization framework is to reduce overfitting by reducing the number of model parameters and the sum of squared errors between the simulated and experimental current-voltage (I–V) curves. In addition to this, authors also explore the difficulties encountered while extracting precise parameters owing to the non-linear and non-unique structure of the I–V curves.

The research by Ibrahim and Anani [10] studies the influence of temperature and irradiance fluctuations on the PV modules' electrical performances, while offering a modified version of the single-diode model. In Ref. [11], five parameters were employed to characterize the electrical behavior of the multi-junction concentrated photovoltaic (CPV) module, and were mainly determined using the multi-dimensional Newton-Raphson approach. The suggested method's lumped parameter values were compared to those obtained using the standard two-diode model. The findings demonstrate that the suggested technique offers more accurate and consistent lumped parameter values than the standard two-diode model. The authors credit the enhanced accuracy to the suggested method's simultaneous consideration of all five parameters, while the standard two-diode model evaluates just two parameters.

The suggested technique in Ref. [12], employs an electrical model of the PV panel that takes into account the solar cell's non-linear and temperature-dependent behavior. The model is used to replicate the solar panel's I–V characteristics, which are then utilized to extract the circuit parameters. The research demonstrates that the derived parameters are compatible with the physical features of the solar cell and can be used to accurately forecast the I–V curve of the solar panel. The strategy may also be used to increase the efficiency of solar systems by optimizing their design. The simulation results indicate that the proposed method can achieve a very high level of accuracy in extracting the equivalent circuit parameters, with a mean absolute percentage error (MAPE) of less than 1% for most parameters. The experimental results show that the proposed method can accurately extract the equivalent circuit parameters under different operating conditions, with a MAPE of less than 1.5% for most parameters.

In [13], a comparative examination of parameter extraction strategies for the electrical characterization of multi-junction concentrator photovoltaic (CPV) and microcrystalline silicon (m-Si) technologies is presented. Authors assess the precision and accuracy of many extraction strategies, including the Lambert W-function, Newton-Raphson method, and Levenberg-Marquardt algorithm. They show that, for both CPV and m-Si devices, the Levenberg-Marquardt method offers the optimal compromise between accuracy and computational efficiency. For the multi-junction CPV cells, one-diode, two-diode, and three-diode models were explored, while for the mono-Si cells, one-diode, two-diode with series resistance, and two-diode with shunt resistance models were considered. The correctness of these models was then determined by comparing the findings derived from these models to experimental data. The scientists discovered that the two-diode model with series resistance offered the greatest match for both multi-junction CPV cells and mono-Si cells.

In [14], an enhanced onlooker-ranking-based adaptive differential evolution (AR-ADE) approach is proposed to correctly extract solar cell model parameters. Experimental data showed that the AR-ADE method can properly extract solar cell model characteristics. According to the paper, the recommended approach extracts parameters more accurately than earlier optimization techniques. Their method extracts a solar cell's single-diode model's features with 99.97% accuracy. Authors further demonstrate that their technique surpasses Particle Swarm Optimization (PSO), Genetic Algorithm (GA), and Differential Evolution (DE) in accuracy and convergence speed. Using assured convergence particle swarm optimization, Nunes et al. [15] have established a novel, high-performance method for determining the parameters of PV cells and modules. The proposed G-CPSO method was compared to four existing contemporary approaches: differential evolution (DE), particle swarm optimization (PSO), adaptive PSO (APSO), and genetic algorithm (GA). In terms of precision and convergence speed, the findings demonstrated that G-CPSO outperformed the other approaches. G-CPSO achieved a 100% convergence rate for both the single diode model and the double diode model, whereas the other techniques achieved a convergence rate between 55% and 85%. In addition, the G-CPSO method had the lowest root mean square error (RMSE) and the highest coefficient of determination (R2) when compared to the other approaches, indicating its superior accuracy in recognizing the properties of PV cells and modules.

In the work presented by Elkholy et Abou El-Ela [16], the single, double and triple diode models were all compared to the suggested analytical model. The comparison was based on the mean absolute percentage error (MAPE) between a photovoltaic module's projected and observed I–V characteristics at various temperatures and irradiance levels. The findings demonstrated that the suggested analytical model, with an average MAPE of 1.86% compared to 4.52%, 4.46%, and 4.59% for the single diode, double diode, and triple diode models, respectively, offered more accurate predictions of the I–V characteristics than the other three models. The suggested analytical model has more accuracy since it accounts for numerous aspects that affect PV module performance, such as temperature, irradiance, and solar radiation spectral distribution. An optimization strategy that calculates ideal values for fewer unknown parameters reduces the model's structure, improving its accuracy. The two-diode model, which includes solar cell recombination mechanisms, also improved the model accuracy.

The study presented by Ma et al. [17] aimed to provide an accurate and comprehensive model for solar PV modules under real-world situations. The authors utilized a single-diode model with series and shunt resistances to characterize the photovoltaic module's I–V and P–V properties. Experimental data from a commercial 200W polycrystalline silicon solar module was used to verify the MATLAB/Simulink model. The suggested model was compared to the single-diode and double-diode models. The maximum power point (MPP) tracking, I–V and P–V characteristics, and simulated and measured outcomes were used to compare. The suggested model demonstrated superior accuracy in estimating MPP, I–V, and P–V characteristics, predicting solar module performance under varied environmental circumstances. The model incorporates temperature and irradiance dependence of photovoltaic module characteristics such diode ideality factor, series and shunt resistance, and open-circuit voltage. The model accounts for parasitic resistances and recombination losses. Simulation results revealed that the proposed model accurately predicted solar module performance under varied environmental circumstances, including irradiance and temperature, outperforming current models in predicting the solar module's MPP and aligning well with experimental data.

In [18], the PV cell is seen as a combination of a current source, a diode, and a resistor in the model, which is based on the equivalent circuit method. Shunt resistance is taken into account by altering the diode equation, and series resistance is represented by an extra resistor in the equivalent circuit. By comparing the predicted results with actual data from several kinds of PV cells, such as crystalline silicon, amorphous silicon, and copper indium gallium selenide (CIGS) cells, the suggested model's accuracy was assessed. With root-mean-square error (RMSE) values ranging from 0.45% to 2.8% for current-voltage (I–V) curves and from 1.8% to 4.3% for power-voltage (P–V) curves, the results demonstrated that the model was capable of accurately predicting the electrical performance of these cells under various operating conditions. A thorough electrical model for PV modules was described in the research by Chennoufi et al. [19]. The model's two-diode foundation allowed for a more realistic description of the modules' electrical behavior, especially at low irradiance levels. The suggested model was tested using experimental data from a commercial PV module, and it was found to produce a good match between the simulated and observed current-voltage (I–V) curves with a high coefficient of determination (R2 = 0.9999). The model's accuracy was shown to be higher, especially in low light settings, when compared to other widely used models like the one-diode and three-diode models. In addition, the suggested model successfully predicted the module's maximum power point and fill factor (FF). The suggested two-diode model may be utilized as an accurate and dependable tool for the design and optimization of PV systems, according to the authors' conclusion.

#### 1.1.2 Thermal modeling

Whether a single-diode, double-diode or triple-diode model is used in estimating PV parameters,the cell's temperature has a high impact on the system's performance. This explains the research focus on it. Skoplaki and Palyvos discussed in Ref. [20] the importance of solar cell operating temperature on the electrical performance of silicon-based PV installation and presented a review of formulations and correlations adequate to different installation situations.

Jones and Underwood [21] developed an analytical thermal model to predict the temperature of photovoltaic cells as a function of environmental conditions. However, due to the assumptions made in their model, in particular the assumption of temperature uniformity throughout the panel, temperature predictions varied by around 6 °C from experimental measurements. Their model showed greater accuracy under clear-sky conditions characterized by more stable irradiance, highlighting a slower response to environmental fluctuations.

Dolara et al. [22] compared three physical models (three, four and five-parameters) for electrical presentation of photovoltaic models while using two different approaches for cell temperature estimation (Sandia and NOCT). These models were tested using 18 PVs, from which ten monocrystalline and eight polycrystalline. Results showed that using complex models is of no benefit. Also, the forecasted power output are accurate using experimental data and Sandia's model for monocrystalline modules. As for the polycrystalline modules, the manufacturer data and nominal operating conditions temperature (NOCT) seemed to be more exact.

In [23], Zhou et al., studied the impact of the highest module temperature with changing back sheet materials on the temperature of a polycrystalline Silicone PV module while keeping constant the Environmental factors. For these purposes, the finite element method using the ANSYS Multiphysics simulator has been used. Results proved that using a thin TPT back sheet can help reduce the model's temperature. However, if the thickness of the back sheet material increases 0.5 mm, the tempered glass or aluminum performs better.

In [24], Nasrin and al., studied the effect of high irradiation on thermal photovoltaics' production through the usage of converging lenses. The study was held in steady state conditions using the COMSOL Multiphysics software, based on the finite element methodology, while considering that the cooling fluid is incompressible and is in laminar conditions. As for the experimental investigation, authors exaggerated it from cell to module level, where each PVT module receives concentrated solar irradiation from concentrating lens. Results showed that increasing irradiation levels contributes to an increase in the cell's temperature as well as output electrical power. As for the overall efficiency, it drops by 0.39% for each 100 W/m^2^ increase of irradiation.

In [25], Du et al., investigated the time dependent thermal behavior of crystalline silicon photovoltaic through the usage of glass-glass and glass-back sheet configurations. The proposed thermal model has been validated using field testing. In fact, results showed that for the GG module with white EVA, the cell's temperature is lower in comparison to the GG module with transparent EVA. However, the GB has the lowest temperature with a protective cover back sheet. Still, the GG configuration showed a better heat dispersion and temperature uniformity than the GB module.

Nasrin et al. [26] studied the effect of using a PVT with Water/(Multi-Walled Carbon Nanotubes) nanofluid on energy production. The study has been held using the COMSOL Multiphysics finite element software and validated through indoor experimental setup, where operating conditions were controlled. Results showed that the performances of the system improved about 9.2%, as for the electrical power, it increased by about 17 W for each 100W/m^2^ increase in irradiation.

In [27], Aly et al. presented a complete detailed step-by-step thermal model enabling the prediction of PV cells temperatures’ as to assess their electrical performances. The model is developed considering transient conditions and can assess any type of free-standing plane PV surfaces. The model has been validated using the NOCT and PVUSA test conditions (PTC) as input parameters. As to back up the results, experimental validation has been held as well in Doha, Qatar for three days under different conditions: summer day with clear sky; winter day with cloudy sky; and spring day with rainy sky. Once validated, the model has been assessed and compared to six empirical thermal models from literature review. Results of these various tests showed that the developed model can accurately capture real thermal behavior of PVs under steady state as well as transient conditions.

In [28], Kirpichnikova et al., developed a thermal model of a photovoltaic module using a heat-protective film based on holographic coating with a total internal reflection prism layer. The model has been simulated on MATLAB/Simulink to calculate the module's temperature and estimate current-voltage and output power-solar irradiance characteristics. The correlation linking between ambient air temperature and module's temperature has been built through the usage of experimentation. This latter's results proved the effect of the film on reducing PV temperature and increasing energy output in arid continental climates. Still, the model needs to be improved to predict temperature and output electrical power in other climates, while accounting for sunlight effect.

Bevilacqua et al., proposed in Ref. [29] a one-dimensional transient thermal model that can estimate PV's module temperature across the thickness of each material's layer and assess its effect on output-power electrical production. The model accounts for ambient air temperature, the temperature at the back of the panel and radiative long wave thermal exchange between the sky and the module. For this, different formulations of heat transfer coefficients were tested to attain more accuracy. The model was validated against experimental data acquired for a whole year, on a seasonal basis, in a test site installed at the University of Calabria, Italy. The model proved its efficiency in terms of estimating temperature but was not as precise in terms of estimating electrical power. From which the necessity of coupling both electrical and thermal models.

Villemin et al. [30], presented a new thermal model based on the Monte Carlo method to estimate the panel's temperature at any probe point, while integrating climatic data. The temperature can be calculated after resolving the system that couples heat equations in transient conditions, boundary conditions of the system as well as initial conditions. As for validation purposes, authors used field testing through the deployment of a 310 W monocrystalline PV. Results of experimentation are in accordance with simulation and the model proved its accuracy in terms of predicting module's temperature as well as estimate annual electrical production.

#### 1.1.3 Coupled electrical and thermal modeling

It is important to understand the existing relationship between electrical and thermal performances of a PV cell. Still, few are the research works that study this area seeing its complexity. Barroso et al. [31] assessed the performances of a commercial PV panel through the coupling of a one-dimensional thermal model based on finite difference approach and an electrical model based on the particle swarm optimization algorithm. For more accuracy, both models were assessed in response to three variable configuration boundary conditions presented by: Notton et al., 2005, Armstrong et Hurley 2010 and Siddiqui et al., 2012. According to Siddiqui et al., the boundary conditions for heat dissipation are convection and radiation from both surfaces (front and rear). As for Notton et al., they based their simulation model on the convective heat transfer coefficient which has different expressions: only the forced convective one, the highest value between the forced and natural coefficient and the sum of both coefficients. Last but not least, Armstrong and Hurley consider the thermal mass of the PV panel, since if neglecting it, any sudden change in the ambient conditions might lead to an abruptly change of the whole PV panel temperature.

Results of all formulations were analyzed using the NOCT conditions, real weather data and a parametric study variating ambient temperature. In Ref. [32], Shang and Li investigated the fundamental physics and light matter interactions in a single-junction Galliun arsenide SC module, through the development of a coupled opto-electro-thermal model developed under COMSOL Multiphysics. The model optically investigates the light absorption, reflection and transmission. It electrically investigates the carrier diffusion, drift and loss mechanisms. As for the thermodynamic investigation, it concerns heat generation and dissipation. Such a developed model allows quantifying energy conversion from light incidence to electricity and thermal energy, which allows a broad range of applications in the design of opto-electronic devices. Cabo et al., focused in their study [33], on the development of a coupled thermo-electrical model in order to assess the performances of free-standing photovoltaic panels exposed to hot-spot effect. This study highlights a new approach based on a two-way coupling regime, namely direct and indirect shadowing. The numerical electrical model was developed using MATLAB as for the thermal model it was developed under ANSYS Fluent and coupled to MATLAB through user defined functions (UDF). The general model has been validated using experimental data acquired by temperature sensors and infra-red camera imaging. Results showed that the model is only accurate for partial and multi hot-spot effects as long as they do not trigger bypass diodes. In Ref. [34], Yaman and Arslan evaluated the performances of their developed step-by-step thermo-electrical model which is used to examine the highest temperature and electrical power consumption for Si-PV under different conditions. Results of simulation were confronted to experimentation and proved the accuracy of the model in terms of accurately estimating electrical and thermal parameters of a PV panel under transient conditions. As to back up the results even more, the model was confronted to some previous models in literature and proved its robustness. Authors in Ref. [35], presented a coupled optical-thermal-electrical model to evaluate output energy production of PV panels, assess the distributed losses and better understand the relationship between electrical production, heat losses and ambient conditions. The model has been evaluated using STC and under dynamic conditions. Results showed that the optical, electrical and thermal losses are, respectively, 16%, 16% and 68%. In order to minimize these losses, especially the thermal ones, some theoretical suggestions have been mentioned to note using down conversion, up conversion, stacked structures and surface recombination.

#### 1.1.4 Optimization algorithms

Over the decades, numerous optimization approaches have been developed to solve a variety of optimization problems. In recent years, however, the complexity of real-world optimization problems has increased considerably, in parallel with the development of human society and modern industrial processes. This poses a growing challenge to optimization techniques. In general, existing optimization techniques can be categorized into two groups: deterministic algorithms and meta-heuristics. Deterministic algorithms are specific mathematical functions that operate mechanically and iteratively, without the introduction of random elements. They always produce the same result for a particular input to a given problem. Classic examples of deterministic algorithms include gradient descent and Newton's methods. While these approaches are effective for finding local optima in solving certain non-linear problems, they may require derived information and be limited in solving complex problems characterized by high constraints and multiple optimal points.

In this context, meta-heuristic methods have become preferred alternatives to deterministic methods. Meta-heuristic methods are attracting considerable attention because of their randomized approach and their distinctive way of dealing with problems. Their randomness reduces sensitivity to initial conditions, facilitating a smooth transition between exploration and exploitation. By adopting a black-box perspective, these methods focus on inputs and outputs rather than on a detailed understanding of problem structure. These strengths give meta-heuristics the ability to efficiently reach global optimal solutions, filling the gaps left by deterministic methods often limited by a lack of derived information or other structured data.

The Genetic Algorithm (GA), one of the classic Evolutionary Algorithms (EA), draws on the mechanisms of natural selection in biological systems to solve complex optimization problems [36]. Its basic version reproduces three key evolutionary behaviors: selection, crossover and mutation. Working with a population of individuals, where each individual represents a candidate solution, the GA evolves this population over time through these three operators. After several iterations, the best individual is used to generate a new population. By selecting individuals in proportion to their fitness values, the GA converges towards the global optimum. Compared with deterministic methods, GA stands out for its intrinsic power and independence from any additional information.

Particle Swarm Optimization (PSO), one of the most widespread bio-inspired methods [37], draws on the social interactions present in bird flocks to solve optimization problems. Initiating the process with a random population of individuals, whose positions represent potential solutions, each iteration sees the stochastic updating of positions according to the best global position of the set of individuals and the best individual position. The fitness function evaluates the quality of each individual.

The Artificial Bee Colony (ABC) algorithm, another prominent example of a bio-inspired approach, is inspired by the intelligent foraging strategies adopted by honey bees [38]. In ABC, three categories of bees are deployed to perform global optimization: employed bees, observer bees and scout bees. Employed bees explore food sources locally based on visual information, observer bees choose a food source with probability proportional to nectar quantity, and scout bees randomly opt for a food source in the search space. Thanks to these varied foraging strategies, the ABC manages to harmonize exploration and exploitation, resulting in rapid convergence.

AHA [39] stands out clearly from existing algorithms as a meta-heuristic, mainly thanks to its unique biological anchoring. It is specifically inspired by three foraging strategies and three flight abilities observed in hummingbirds. A major difference lies in the balance between exploration and exploitation, where migration favors exploration, territory foraging favors exploitation, and guided foraging favors early exploration and later exploitation. A third distinctive feature is the specific memory updating mechanism, involving the recording of reciprocal hummingbird visits in a table. In this way, AHA differs significantly from existing algorithms, aspiring to mimic the complex foraging behaviors of hummingbirds, with particular attention paid to their exceptional memory and flight skills.

### 1.2 Limitations and research gap

As stated previously, electrical methods adapted for PV performance evaluation and modeling are various, as can be cited: Empirical Formula (EF), Single-Diode Model (SDM), Double-Diode Model (DDM) and Multi-Diode Model (MDM). Even if most of them have a broad applicability, still they have many disadvantages. Starting with the EF, it is the least accurate method among them all as it is unable to simulate the real behavior of PVs. Moving to the SDM, DDM and the MDM, they become more accurate as the number of diodes increases, still the process becomes much more complex and is highly time consuming. As for the thermal sub-models, most of the existing ones have low accuracy as they do not consider the ambient factors and their applicability is limited to one dimensional case. Considering the coupled models, difficulties in coupling are encountered, the computational cost is high and their applicability is barely centered around engineering problems.

### 1.3 Objectives of the study

In this research, an improved holistic model combining both electrical and thermal modeling of a PV system will be presented. The main objective of this proposed model is to be, simultaneously, able to estimate the electrical characteristics as well as the cell's temperature distribution. The electrical sub-model is based on the use of a double-diode configuration, with a robust algorithm for parameter extraction; namely the Metaheuristic Algorithm (MA). As for the thermal sub-model, it is based on finite differences and is developed and assessed using various convective heat transfer coefficients for enhanced accuracy. The physical model and its related mathematical formulations are presented. The electrical, thermal and coupled models will be run using Python. The effectiveness of the proposed models is assessed by comparing them to real time experimental data.

## 2 Materials and methods

### 2.1 Electrical sub-model

Analyzing the operation of a PV system and predicting the impact of various factors on the behavior of a PV module, particularly in changing environmental conditions, is highly dependent on the proper modeling and design of the PV module.

#### 2.1.1 Mathematical equations of DDM

**Fig. 1:**
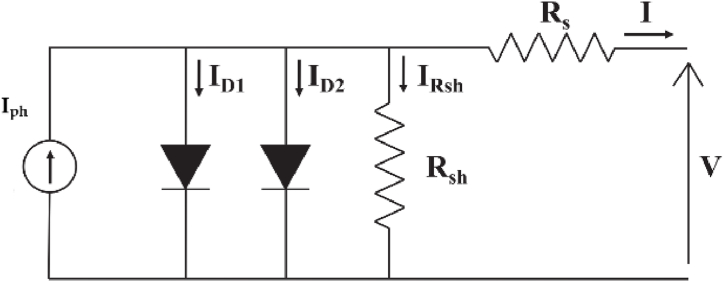
Equivalent circuit adopting double-diode model of a PV module.

To enhance the precision of the electrical model, a Double-Diode lumped parameter model, depicted in Fig. 1, has been adopted. The double-diode PV model features a solar cell represented by a current source, which is connected in parallel with two distinct diodes (D1 and D2), along with a shunt resistance (R_sh_). All of these components are connected in series with a series resistance (R_s_). The inclusion of another diode in the single-diode PV model is an illustration of recombination occurring in the depletion region.

Applying Kirchhoff's current law results in the mathematical expression, for the double-diode model, expressed by Eq. (1) as follows:

(1) $$I=I_{ph}-I_{D1}-I_{D2}-I_{R_{sh}}$$

Adopting the well-known Shockley diode equation, the current through the two diodes (D1 and D2) can be determined. Consequently, the current-voltage relationship for the double-diode model can be expressed by Eq. (2) as follows:

(2) $$I=I_{ph}-I_{s1}\left(e^{\frac{V+IR_{s}}{{a_{1}Ns\;V}_{T}}}-1\right)-I_{s2}\left(e^{\frac{V+IR_{s}}{{a_{2\;}Ns\;V}_{T}}}-1\right)-\left(\frac{V+IR_{s}}{R_{sh}}\right)$$

where I_ph_ is the current generated by the solar irradiance, (a_1_, a_2_) and (Is1 and I_s2_) are the ideality factors and reverse saturation currents of the first and second diode respectively. The series and parallel resistances can be represented as (R_s_ and R_sh_), respectively. V_T_ (=KT)/q) is the thermal voltage of the PV module where K is the Boltzmann constant ($1.3806503\times 10^{-23}$ J/K), q is electron charge ($1.60217646\times 10^{-19}$ C) and T is the temperature in K. The variable Ns relates to the number of cells that are linked in series within the PV module.

There are seven unknown parameters involved in Eq. (1), a_1_, a_2_, I_s1_, I_s2_, R_s_, R_sh_ and I_ph_.

A set of seven nonlinear equations is formulated to calculate these seven parameters.

At open circuit point (V=V_oc,_ I = 0), Based on Eq. (1), we end up with Eq. (3) below:

(3) $$0=I_{ph}-I_{s1}\left(e^{\frac{V_{oc}}{{a_{1}Ns\;V}_{T}}}-1\right)-I_{s2}\left(e^{\frac{V_{oc}}{{a_{2}Ns\;V}_{T}}}-1\right)-\left(\frac{V_{oc}}{R_{sh}}\right)$$

At short circuit point (V = 0, I=I_sc_) drawing on Eq. (1), we end up with Eq. (4) below:

(4) $$I_{sc}=I_{ph}-I_{s1}\left(e^{\frac{I_{sc}R_{s}}{{a_{1}Ns\;V}_{T}}}-1\right)-I_{s2}\left(e^{\frac{I_{sc}R_{s}}{{a_{2}Ns\;V}_{T}}}-1\right)-\left(\frac{I_{sc}R_{s}}{R_{sh}}\right)$$

At maximum power point (V=V_mp_,I=I_mp_), based on Eq. (1) we obtain equation (5) expressed as follows:

(5) $$I_{mp}=I_{ph}-I_{s1}\left(e^{\left(\frac{V_{mp}+I_{mp}R_{s}}{{a_{1}Ns\;V}_{T}}\right)}-1\right)-I_{s2}\left(e^{\left(\frac{V_{mp}+I_{mp}R_{s}}{{a_{2}Ns\;V}_{T}}\right)}-1\right)-\left(\frac{V_{mp}+I_{mp}R_{s}}{R_{sh}}\right)$$

The power output of a PV module at any point on the I–V curve is given by P=VI. The derivative of power with respect to voltage is $\frac{dP}{dV}=I+V\frac{dI}{dV}$. However, at the maximum power point (MPP), the derivative of power with respect to voltage is zero i.e., $\frac{dP}{dV}=0$ which occurs at the standard test conditions (STC) and can be written as shown below on Eq. (6):

(6) $$\frac{dI}{dV}=-\frac{I_{mp}}{V_{mp}}$$

To obtain the term $\frac{dI}{dV}$ , Eq. (5) is differentiated with respect to voltage, resulting in Eq. (7) expressed as follows:

(7) $$\frac{dI}{dV}=\frac{\frac{-I_{s1}}{a_{1}Ns\;V_{T}}e^{\left(\frac{V+R_{s}I}{a_{1}Ns\;V_{T}}\right)\;}-\frac{I_{s2}}{a_{2}Ns\;V_{T}}e^{\left(\frac{V+R_{s}I}{a_{2}Ns\;V_{T}}\right)\;}-\frac{1}{R_{sh}}}{1+\frac{R_{s}I_{s1}}{a_{1}Ns\;V_{T}}e^{\left(\frac{V+R_{s}I}{a_{1}Ns\;V_{T}}\right)\;}+\frac{R_{s}I_{s2}}{a_{2}Ns\;V_{T}}e^{\left(\frac{V+R_{s}I}{a_{2}Ns\;V_{T}}\right)\;}+\frac{R_{s}}{R_{sh}}}$$

By substituting Eq. (6) into Eq. (7), Eq. (8) is derived:

(8) $$\frac{\frac{-I_{s1}}{a_{1}Ns\;V_{T}}e^{\left(\frac{V_{mp}+R_{s}I_{mp}}{a_{1}Ns\;V_{T}}\;\right)\;}-\frac{I_{s2}}{a_{2}Ns\;V_{T}}e^{\left(\frac{V_{mp}+R_{s}I_{mp}}{a_{2}Ns\;V_{T}}\right)\;}-\frac{1}{R_{sh}}}{1+\frac{R_{s}I_{s1}}{a_{1}Ns\;V_{T}}e^{\left(\frac{V_{mp}+R_{s}I_{mp}}{a_{1}Ns\;V_{T}}\right)\;}+\frac{R_{s}I_{s2}}{a_{2}Ns\;V_{T}}e^{\left(\frac{V_{mp}+R_{s}I_{mp}}{a_{2}Ns\;V_{T}}\right)\;}+\frac{R_{s}}{R_{sh}}}=-\frac{I_{mp}}{V_{mp}}$$

The derivative of current with respect to voltage at open circuit conditions is $\frac{dI}{dV}=-\frac{1}{R_{s}}$ (9), substituting these values into Eq. (7), one can write Eq. (10) expressed as follows:

(10) $$\frac{\frac{-I_{s1}}{a_{1}Ns\;V_{T}}e^{\left(\frac{V_{oc}}{a_{1}Ns\;V_{T}}\right)\;}-\frac{I_{s2}}{a_{2}Ns\;V_{T}}e^{\left(\frac{V_{oc}}{a_{2}Ns\;V_{T}}\right)\;}-\frac{1}{R_{sh}}}{1+\frac{R_{s}I_{s1}}{a_{1}Ns\;V_{T}}e^{\left(\frac{V_{oc}}{a_{1}Ns\;V_{T}}\right)\;}+\frac{R_{s}I_{s2}}{a_{2}Ns\;V_{T}}e^{\left(\frac{V_{oc}}{a_{2}Ns\;V_{T}}\right)\;}+\frac{R_{s}}{R_{sh}}}=-\frac{1}{R_{s}}$$

The derivative of current with respect to voltage at short circuit conditions is

(11) $$\frac{dI}{dV}=-\frac{1}{R_{sh}}$$

substituting these values into Eq. (7), one can obtain Eq. (12):

(12) $$\frac{\frac{-I_{s1}}{a_{1}Ns\;V_{T}}e^{\left(\frac{R_{s}I_{sc}}{a_{1}Ns\;V_{T}}\right)\;}-\frac{I_{s2}}{a_{2}Ns\;V_{T}}e^{\left(\frac{R_{s}I_{sc}}{a_{2}Ns\;V_{T}}\right)\;}-\frac{1}{R_{sh}}}{1+\frac{R_{s}I_{s1}}{a_{1}Ns\;V_{T}}e^{\left(\frac{R_{s}I_{sc}}{a_{1}Ns\;V_{T}}\right)\;}+\frac{R_{s}I_{s2}}{a_{2}Ns\;V_{T}}e^{\left(\frac{R_{s}I_{sc}}{a_{2}Ns\;V_{T}}\;\right)\;}+\frac{R_{s}}{R_{sh}}}=-\frac{1}{R_{sh}}$$

Typically, it is observed that the magnitude of I_s2_ is approximately three to four times larger than I_s1_ [40], and it can be considered as written on Eq. (13):

(13) $$I_{s2}=\left(\frac{T^{\frac{2}{5}}}{3.77}\right)I_{s1}$$

Now, the seven non-linear equations have been formulated in order to extract the unknown parameters.

#### 2.1.2 Artificial hummingbird algorithm

Based on the results of a previous study conducted by Navarro et al. [41], which compared the performance of eight recently proposed metaheuristic algorithms in optimizing solar cell parameters, the Artificial Hummingbird Algorithm (AHA) [39] was found to have the highest performance and resulted in minimal errors. Therefore, in the current paper, the AHA algorithm was selected as the most suitable choice among the eight algorithms studied for parameter extraction that will be included in the DDM. The AHA algorithm is a metaheuristic algorithm known for its ability to efficiently explore the search space and find optimal solutions by mimicking the foraging behavior of hummingbirds. The Artificial Hummingbird Algorithm (AHA) was introduced in 2022 by Zhao, Wang and Mirjalili [39], drawing inspiration from the intelligence and foraging behavior of hummingbirds. The algorithm employs three basic concepts: food sources, hummingbirds, and a table to keep track of food source visits. During the search process, each agent evaluates the food sources based on their recharge rate and can remember and share the position of each food source with other agents. Additionally, hummingbirds can recall the time elapsed since they last visited a particular food source. Eq. (14) is used to determine the new positions for each hummingbird. The model of this behavior is presented on Eq. (15). The authors also developed mathematical models for three types of flight: axial flight Eq. (16), diagonal flight Eq. (17), and omnidirectional flight Eq. (18), which introduce a vector of change for individual positioning.

(14) $$x_{i}\left(t+1\right)=\left\{ \begin{array}{c} x_{i}\left(t\right)\;f\left(x_{i}\left(t\right)\right)\leq f\left(v_{i}\left(t+1\right)\right)\\ v_{i}\left(t+1\right)\;f\left(x_{i}\left(t\right)\right)>f\left(v_{i}\left(t+1\right)\right) \end{array}\right.$$

(15) $$v_{i}\left(t+1\right)=x_{i,tar}\left(t\right)+N\left(0,1\right).D.\left(x_{i}\left(t\right)-x_{i,tar}\left(t\right)\right)$$

(16) $$D^{\left(i\right)}=\left\{ \begin{array}{c} 1,if\;i=\text{randi}\left(\left[1,d\right]\right)\;\\ 0,else \end{array}\right.\;i=1,2,3,\ldots \ldots \ldots \ldots \ldots .d$$

(17) $$D^{\left(i\right)}=\left\{ \begin{array}{c} 1,i=P\left(j\right),j\in \left[1,k\right],P=\text{randperm}\left(k\right),k\in \left[2,\lceil r_{1}\;\cdot \;\left(d\;-\;2\right)\rceil +1\right]\;\\ 0,else \end{array}\right.i=1,2,3,\ldots \ldots \ldots \ldots \ldots .d$$

(18) $$D^{\left(i\right)}=1i=1,\ldots .d$$

where $x_{i}$ represents the position of the ith food source that is the solution of a given problem, d-dimensional problem.

**Fig. 2:**
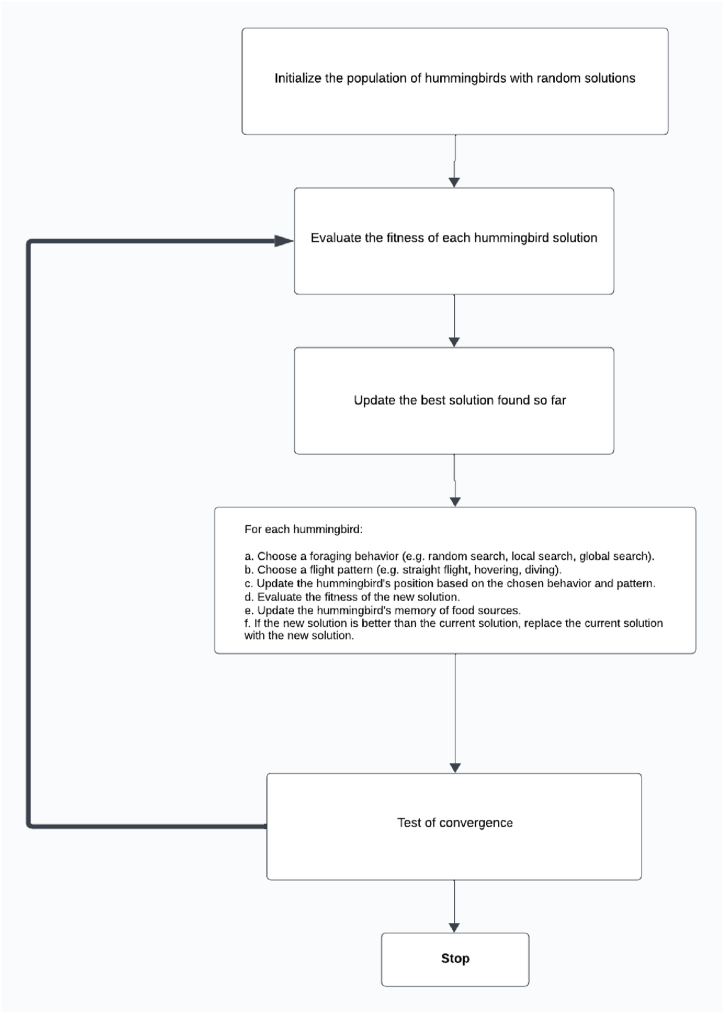
Flowchart of AHA algorithm [39].

The flowchart of the AHA algorithm, presented in Fig. 2, provides a clear and structured overview of the algorithm's processes, including the initialization of the population, evaluation of fitness, foraging behaviors, flight patterns, and memory updates.

The implementation of the AHA algorithm was carried out in MATLAB 2022a. A population size of 50 was chosen, as it has been shown to yield better performance with the AHA algorithm [39].To account for the presence of a larger number of computed variables and the complex nature of the objective function, a maximum number of 2000 iterations was set. This was done to ensure that the algorithm converged to a satisfactory solution within the given constraints. Table 1 provides a summary of the inputs that are specified by the user.

**Table 1:** User defined inputs.

Parameters	Values
The maximum number of iterations	2000
The size of hummingbird population	50
The dimensionality of problem	7
The low boundary of search space	[0.5, 0.5, 10^−12^, 10^−12^, 0.01, 100]
The up boundary of search space	[3, 3, 10^−3^, 10^−3^, 1, 2000]

#### 2.1.3 The formulation of the objective function

The primary goal of optimization in this study is to accurately estimate the seven unknown parameters of the PV module, mainly a_1_, a_2_, I_s1_, I_s2_, R_s_, R_sh_ and I_ph_. The parameters must be estimated in such a way that the parameter values presented in Table 2 are satisfied. Achieving such a thing would ensure that the PV module operates at its maximum efficiency, which is crucial for its practical applications. Accurate estimation of the PV module parameters is also essential for the development of effective control strategies and for predicting the behavior of the cell under different operating conditions.

**Table 2:** Electrical properties of the different PV panels’ technologies.

Parameters	Mono-crystalline SM55	Multi-crystalline S75	Thin film ST36
**I**_**SC**_**(A)**	3.45	4.70	2.68
**V**_**OC**_**(V)**	21.7	21.6	22.9
**I**_**MP**_**(A)**	3.15	4.26	2.28
**V**_**MP**_**(V)**	17.4	17.6	15.8
**K**_**V**_**(mV/°C)**	−76	−76	−100
**K**_**I**_**(mA/°C)**	1.4	2	0.32
**N**_**s**_	36	36	42

Based on the mathematical equations presented for the double diode model, seven objective functions will be generated (Eq 19-Eq (25)). The sum of the squares functions (also named as error functions) will be deployed to estimate the overall error of the system, which must be near zero for a better performance. In other words, the overall error (Eq. (26)) serves as a mathematical representation of the optimization problem and is used to evaluate the fitness of potential solutions. Giving the data presented in Table 2, the optimal solution shall enable the estimation of these parameters at STC conditions and yields the best possible estimation of the PV cell parameters (current, voltage and power).

Based on Eq. (3), the error function at open circuit point is set as:

(19) $$f_{1}=I_{ph}-I_{s1}\left(e^{\left(\frac{V_{oc}}{{a_{1}Ns\;V}_{T}}\right)}-1\right)-I_{s2}\left(e^{\left(\frac{V_{oc}}{{a_{2}Ns\;V}_{T}}\right)}-1\right)-\left(\frac{V_{oc}}{R_{sh}}\right)$$

Based on Eq. (4), the error function at short circuit point is set as:

(20) $$f_{2}=I_{ph}-I_{s1}\left(e^{\left(\frac{I_{sc}R_{s}}{{a_{1}Ns\;V}_{T}}\right)}-1\right)-I_{s2}\left(e^{\left(\frac{I_{sc}R_{s}}{{a_{2}Ns\;V}_{T}}\right)}-1\right)-\left(\frac{I_{sc}R_{s}}{R_{sh}}\right)-I_{sc}$$

Based on Eq. (5), the error function at MPP is set as:

(21) $$f_{3}=I_{ph}-I_{s1}\left(e^{\left(\frac{V_{mp}+I_{mp}R_{s}}{{a_{1}Ns\;V}_{T}}\right)}-1\right)-I_{s2}\left(e^{\left(\frac{V_{mp}+I_{mp}R_{s}}{{a_{2}Ns\;V}_{T}}\right)}-1\right)-\left(\frac{V_{mp}+I_{mp}R_{s}}{R_{sh}}\right)-I_{mp}$$

Based on Eq. (8), the error function at MPP is set as:

(22) $$f_{4}=\frac{\frac{-I_{s1}}{a_{1}Ns\;V_{T}}e^{\left(\frac{V_{mp}+R_{s}I_{mp}}{a_{1}Ns\;V_{T}}\right)\;}-\frac{I_{s2}}{a_{2}Ns\;V_{T}}e^{\left(\frac{V_{mp}+R_{s}I_{mp}}{a_{2}Ns\;V_{T}}\right)\;}-\frac{1}{R_{sh}}}{1+\frac{R_{s}I_{s1}}{a_{1}Ns\;V_{T}}e^{\left(\frac{V_{mp}+R_{s}I_{mp}}{a_{1}Ns\;V_{T}}\;\right)\;}+\frac{R_{s}I_{s2}}{a_{2}Ns\;V_{T}}e^{\left(\frac{V_{mp}+R_{s}I_{mp}}{a_{2}Ns\;V_{T}}\right)\;}+\frac{R_{s}}{R_{sh}}}+\frac{I_{mp}}{V_{mp}}$$

Based on Eq. (10), the error function at open circuit point is set as:

(23) $$f_{5}=\frac{\frac{-I_{s1}}{a_{1}Ns\;V_{T}}e^{\left(\frac{V_{oc}}{a_{1}Ns\;V_{T}}\right)\;}-\frac{I_{s2}}{a_{2}Ns\;V_{T}}e^{\left(\frac{V_{oc}}{a_{2}Ns\;V_{T}}\right)\;}-\frac{1}{R_{sh}}}{1+\frac{R_{s}I_{s1}}{a_{1}Ns\;V_{T}}e^{\left(\frac{V_{oc}}{a_{1}Ns\;V_{T}}\right)\;}+\frac{R_{s}I_{s2}}{a_{2}Ns\;V_{T}}e^{\left(\frac{V_{oc}}{a_{2}Ns\;V_{T}}\right)\;}+\frac{R_{s}}{R_{sh}}}+\frac{1}{R_{s}}$$

Based on Eq. (12), the error function at short circuit point is set as:

(24) $$f_{6}=\frac{\frac{-I_{s1}}{a_{1}Ns\;V_{T}}e^{\left(\frac{R_{s}I_{sc}}{a_{1}Ns\;V_{T}}\right)\;}-\frac{I_{s2}}{a_{2}Ns\;V_{T}}e^{\left(\frac{R_{s}I_{sc}}{a_{2}Ns\;V_{T}}\right)\;}-\frac{1}{R_{sh}}}{1+\frac{R_{s}I_{s1}}{a_{1}Ns\;V_{T}}e^{\left(\frac{R_{s}I_{sc}}{a_{1}Ns\;V_{T}}\right)\;}+\frac{R_{s}I_{s2}}{a_{2}Ns\;V_{T}}e^{\left(\frac{R_{s}I_{sc}}{a_{2}Ns\;V_{T}}\right)\;}+\frac{R_{s}}{R_{sh}}}+\frac{1}{R_{sh}}$$

Based on Eq. (13), the error function relates to assumption of magnitude of the reverse saturations current ca be expressed as follows:

(25) $${f_{7}=I}_{s2}-\left(\frac{T^{\frac{2}{5}}}{3.77}\right)I_{s1}$$

The sum of squared errors is used as the global optimization function, meaning that the algorithm should aim to achieve a minimum value of zero for the overall error defined as:

(26) $${{{{F=f}_{1}}^{2}{{+f}_{2}}^{2}+f}_{3}}^{2}+{f_{4}}^{2}+{f_{5}}^{2}+{f_{6}}^{2}+{f_{7}}^{2}$$

#### 2.1.4 Parameters at real outdoor conditions

Given the fact that most PV modules operate in outdoor settings where the panels are exposed to environmental conditions that differ from STC, the unknown parameters are affected by factors such as solar radiation and temperature. Therefore, it is crucial to adjust these parameters to match real outdoor conditions by utilizing the equations provided below [4].

The I_ph_ can be calculated for any given temperature and irradiance adopting Eq. (27) shown below:

(27) $$I_{ph}=I_{ph,STC}\frac{G}{G_{STC}}\left[1+K_{I}\left(T-T_{STC}\right)\right]$$

Whereas G represents the solar irradiance under outdoor conditions, G_STC_ and T_STC_ denotes the solar irradiance and temperature under standard conditions, and k_i_ is the current temperature coefficient of PV module.

The equations below (Eqs. (28), (29))) are used to determine the reverse saturation currents under outdoor conditions:

(28) $$I_{s1}=I_{s1,STC}{\left\lceil \frac{T}{T_{STC}}\right\rceil }^{3}e^{\left(\frac{q}{a_{1}K}\right)\left(\frac{E_{g,STC}}{T_{STC}}-\frac{E_{g}}{T}\right)}$$

(29) $$I_{s2}=I_{s2,STC}{\left\lceil \frac{T}{T_{STC}}\right\rceil }^{3}e^{\left(\frac{q}{a_{2}K}\right)\left(\frac{E_{g,STC}}{T_{STC}}-\frac{E_{g}}{T}\right)}$$

The bandgap energy can be expressed as presented on Eq. (30) below:

(30) $$E_{g}=E_{g,STC}\left(1-0.0002677\left(T-T_{STC}\right)\right)$$

The values of series and shunt resistances are provided in Eqs. (31) and (32)

(31) $$R_{s}=R_{s,STC}\frac{T}{T_{STC}}\lceil 1-0.217In\left(\frac{G}{G_{STC}}\right)\rceil$$

(32) $$R_{sh}=R_{sh,STC}\frac{G_{STC}}{G}$$

This study assumes that the ideality factors have negligible changes with respect to temperature and irradiance [42,43].

#### 2.1.5 Implementation

The electrical model has been implemented into Python, following the procedure bellow.

%9. Define the PV module data sheet.
%9. Build the objective function based on equations at the key points.
%9. Use the metaheuristic algorithm AHA to solve the equation for the objective function.
%9. After determining the values at STC, utilize formulae to determine the parameters at general conditions.
%9. Calculate the current, voltage, and power once the parameters have been established.

### 2.2 Thermal sub-model

#### 2.2.1 Solar radiation

As it is commonly known, not all incident solar radiation on the surface of the PV panel reaches the cell as it is. In fact, a portion is firstly reflected by the front glass, as for the rest, it is absorbed by the different layers before reaching the cell. In order to reduce these kinds of losses, especially the optical ones, many manufacturers opted for the usage of anti-reflecting coating.In order to correctly estimate the output electric power of the PV panels, it is important to accurately calculate the total absorbed solar radiation from the total incident solar radiations. In literature, models vary from simple to improved ones. In this work, an expression (see Eq. (33)) based on the ASHRAE convention is adopted [44]:

(33) $$SR={\left(\tau_{g}\alpha_{PV}\right)}_{n}\left[\left({GF}_{b}∙G_{b}∙{AM}_{b}\right)+\left(G_{d}∙{AM}_{d}∙\frac{1+\cos\left(\beta \right)}{2}\right)+\left(\left(G_{b}+G_{d}\right)∙alb∙{AM}_{gr}∙\frac{1-\cos\left(\beta \right)}{2}\right)\right]$$

where τ is the transmissivity, α is the absorptivity, GF is the geometric factor, G is the solar irradiance on a horizontal plane, AM is the incident angle modifier, β is the tilt angle of the panel and alb is the albedo. As for the subscripts, n stands for normal, b for beam and d for diffuse.

The geometric factor (${GF}_{b})$ in Eq. (33) represents the ratio between beam radiation on a tilted surface and beam radiation on a horizontal surface as expressed on Eq. (34).

(34) $${GF}_{b}=\frac{G_{b,T}}{G_{b,H}}=\frac{\cos\;\left(\theta_{b}\right)}{\cos\;\left(\theta_{z}\right)}$$

where $\theta_{b}$ and $\theta_{z}$ are respectively the angle of incidence of the beam radiation and the sun zenith angle. The subscripts b,TandH are respectively, beam, tilt and horizontal.

The incident angle modifier factors in Eq. (33) can be calculated based on Eq. (35) presented below:

(35) $${AM}_{i}=1+b_{0}\left(\frac{1}{\cos\left(\theta_{i}\right)}-1\right)$$

Where the subscript i can either be: b for beam, d for diffuse or gr for ground. As for $b_{0}$, it is a constant taken as −0.1, as suggested by Notton et al. [45]. It is also worth noting that the expression of the incident angle modifier is only valid when the angle is lower than 60° [44]. Still in the work presented by Ref. [46], the expressions were kept as valid even when the angle was greater than 60°, and advised to suppress it if the angle is too close from 90°.

The angle of incidence for beam irradiance can be calculated using Eq. (36)

(36) $$\theta_{b}=\cos^{-1}\left(\left(\cos\left(\theta_{z}\right)\cos\left(\beta \right)\right)+\left(\sin\left(\theta_{z}\right)\theta_{z}\;\sin\left(\beta \right)\beta \;\cos\;\left(\gamma_{s}-\gamma \right)\right)\right)$$

where $\gamma_{s}$ is the solar azimuth angle while γ is the surface azimuth angle assumed as zero.

The beam radiation on a horizontal surface can be calculated based on Eq. (37) as follows:

(37) $$\cos\left(\theta_{z}\right)=\sin\left(\alpha_{s}\right)=\left(\sin\left(\varphi \right)\varphi \;\sin\left(\delta \right)\right)+\left(\cos\left(\varphi \right)\varphi \;\cos\left(\delta \right)\delta \;\cos\left(h\right)h\right)$$

where $\alpha_{s}$ is the solar altitude, φ is the latitude, δ is the sun declination and h is the local solar time.

The solar azimuth angle can be calculated using Eq. (38) shown below

(38) $$\sin\left(\gamma_{s}\right)=\frac{\sin\left(h\right)\times \cos\left(\delta \right)}{\sin\left(\theta_{z}\right)}$$

As for the angles of incidence for respectively, diffuse and ground, they can be approximated as shown in Eqs. (39), (40) [47]:

(39) $$\theta_{d}=59.68-0.1388\beta +0.001497\beta^{2}$$

(40) $$\theta_{gr}=90-0.5788\beta +0.002693\beta^{2}$$

The optical model presented in Eq. (34), (35), (36), (37), (38), (39), (40), (41) are only valid when the plane-of-array irradiation (G_POA_) is not available. As for our case, and since the G_POA_ is known since it is directly measured from the field weather station, the total absorbed solar radiation can be calculated as follows:

(41) $$S=\alpha_{PV}\times \tau \left(\theta_{b}\right)\times G_{POA}$$

τ is the transmittivity of the glass for the beam component of the irradiation angle of incidence of $\theta_{b}$, $\alpha_{PV}$ is the absorptivity of the PV (=0.93 [48]). The transmittivity can be expressed as shown on Eq. (42)

(42) τ(θb)=e−(kefgcos(θr))[1−12(sin2(θr−θb)sin2(θr+θb)+tan2(θr−θb)tan2(θr+θb))]

K is the extinction constant of the front glass (for most panels, the typical value is 4 m^−1^), $e_{fg}$ is the thickness of the front glass, $\theta_{r}$ is the angle of refraction and can be found using the law of Snell [49] expressed in Eq. (43):

(43) $$\theta_{r}=\sin^{-1}\left(\frac{n_{1}}{n_{2}}\sin\left(\theta_{b}\right)\right)$$

where $n_{1}=$ 1 is the refractive index of air, $n_{2}=$ 1.526 is the refractive index of the front glass. $\theta_{b}$ can be calculated from Eq. (36).

#### 2.2.2 Thermal modeling

**Fig. 3:**
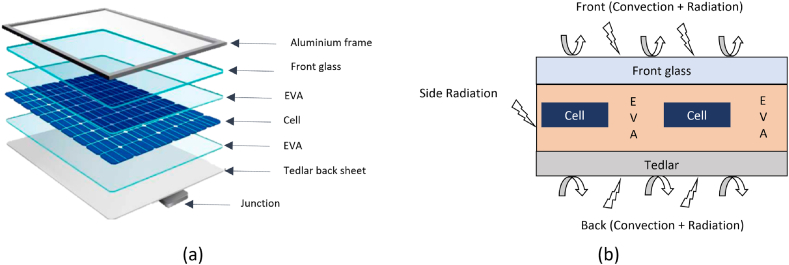
Thin-film PV cell: (a) structure and layering (b) thermal exchanges.

In this study, a thin-film PV system of 5056 mm, from MITSUBISHI ELECTRIC [50] has been used. Each of these panels is made from six layers in the following order: anti-reflecting coating, front glass cover, EVA binder, PV cells, EVA binder and Tedlar back sheet. For simplification purposes, since the anti-reflecting coat has very small thickness and barely any thermal resistance, it has been omitted from this study. Furthermore, the back contact of the cells was omitted as well as the Aluminum frame enclosing the structure. Fig. 3 presents the schematic cross-section of the PV panel; Fig. 3 (a) presents the structure and layering of the PV panel as for Fig. 3 (b) it presents the different thermal exchanges. Moreover, the thermal properties of the layers are enlisted in Table 3.

**Table 3:** Thermal properties of the PV panel construction materials.

Material	Thickness (m)	Thermal conductivity (W∙m^−1^∙K^−1^)	Density (Kg∙m^−3^)	Specific heat capacity (J∙Kg^−1^∙K^−1^)
**Glass**	$3\times 10^{-3}$	1.8	3000	500
**EVA**^a^	$2\times 10^{-2}$	0.35	960	2090
**PV Cell**	$3\times 10^{-2}$	148	2330	677
**Tedlar**	$10^{-2}$	0.2	1200	1250

a The EVA figures as two layers, one of top and one bellow the PV cell, with identical properties.

#### 2.2.3 Heat equation

In this work, a 2-D thermal model is proposed to calculate the generated heat by the PV cells, which is then conducted to the surrounding layers before it being vented out through convection and radiation phenomena.The basic heat equation in 3-D is given as expressed on Eq. (44) below:

(44) $$\rho_{mat}C_{mat}\frac{\partial T\left(x,y,z,t\right)}{\partial t}=\lambda \left(\frac{\partial^{2}T\left(x,y,z,t\right)}{\partial^{2}x}+\frac{\partial^{2}T\left(x,y,z,t\right)}{\partial^{2}y}+\frac{\partial^{2}T\left(x,y,z,t\right)}{\partial^{2}z}\right)+\dot{Q}$$

where ρ is the density of the material, C is the thermal capacity of the material, t is the time, (x,y,z) are space coordinates, T(x,y,z,t) is the temperature spatial distribution at a given time as for $\dot{Q}$ it presents the internal heat generation.

In our case, since the model is 2-D, Eq. (44) can be reformulated into Eq. (45):

(45) $$\rho_{mat}C_{mat}\frac{\partial T\left(x,y,t\right)}{\partial t}=\lambda \left(\frac{\partial^{2}T\left(x,y,t\right)}{\partial^{2}x}+\frac{\partial^{2}T\left(x,y,t\right)}{\partial^{2}y}\right)+\dot{Q}$$

#### 2.2.4 Internal heat generation

Part of the incident solar radiation is reflected by the glass, the rest is absorbed by the front glass and then transmitted to the PV cell while going through the different layers.

The heat generated inside the front glass can be expressed following Eq. (46):

(46) $${\dot{Q}}_{fg}=\frac{\alpha_{fg}\times S_{PV,panel}\times G_{PV}}{V_{fg}}$$

where $G_{PV}$ is plane of array irradiance, $S_{PV,panel}$ is the total surface of the PV panel, $V_{fg}$ is the total volume of the front glass, $\alpha_{fg}$ (=0.05 [51]) is the absorptivity of the front glass. As for the subscript fg, it refers to front glass.

A portion of the solar radiation absorbed by the PV cell, is not entirely converted into electricity, but, is converted into heat and arises cells’ temperature. This internally generated heat can be assumed using Eq. (47) expressed below:

(47) $${\dot{Q}}_{PV}=\frac{SR\times S_{PV,cells}\times \left[1-\left(\eta_{PV,Tref}\left(1-\beta_{ref}\left(T_{PV,cell}-T_{ref}\right)\right)\right)\right]}{V_{PV,cell}}$$

where SR is the solar radiation, $S_{PV,cells}$ is the cumulative surface of the PV cells, $V_{PV,cell}$ is the total volume of the PV cells, $\beta_{ref}$ is an electronic property dependent on the temperature, $T_{ref}$ is the reference temperature (usually 25 °C), $\eta_{PV,Tref}$ is the panel's electrical efficiency at reference temperature. It is to note that both $\beta_{ref}$ (expressed on Eq. (48)) and $\eta_{PV,Tref}$ values figure on the data sheet of the panel [51].

(48) $$\beta_{ref}=\frac{1}{T_{High}-T_{ref}}$$

where $T_{High}$ is the highest temperature at which the Panel's efficiency becomes zero.

#### 2.2.5 Heat exchange

In the present work, all three mode of heat transfer are considered while studying the PV panel: conduction through the materials, convection with the surrounding air and radiation with both ambient air and ground.

In order to correctly model the exchange at the top and bottom surfaces of the PV panels, it is crucial to set appropriate boundary conditions for both convective and radiative exchanges. More details about correlations may be found in Appendix A.

#### 2.2.6 Radiative heat exchange

In this research work, the radiative heat exchange is made through two contributions, radiative between the PV and the sky and between the PV and the ground.

The radiative heat losses from front or back surfaces are given using Eq. (49). Where the convective heat transfer coefficients are expressed using Eq. (50), (51)

(49) $$q_{rad}=h_{rad,surf-sky/gr}\left(T_{surf}-T_{amb}\right)$$

(50) $$h_{rad,surf-sky}=\frac{\sigma ∙\left(T_{surf}^{2}+T_{sky}^{2}\right)\left(T_{surf}+T_{sky}\right)}{\frac{1-\varepsilon_{surf}}{\varepsilon_{surf}}+\frac{1}{F_{surf-sky}}}$$

(51) $$h_{rad,surf-gr}=\frac{\sigma ∙\left(T_{surf}^{2}+T_{gr}^{2}\right)\left(T_{surf}+T_{gr}\right)}{\frac{1-\varepsilon_{surf}}{\varepsilon_{surf}}+\frac{1}{F_{surf-gr}}}$$

where σ is the Stefan-Boltzmann constant (=5.67 × 10^−8^), T is the temperature, ε is the material's emissivity, F is the view factor from any panel surface to the sky or ground (see Appendix A). As for the subscripts surf and gr, are respectively, the surface (front or back) and ground.

$\varepsilon_{fg}=0.91\;\varepsilon_{Td}=0.85$ [49]

The sky temperature can be approximated using Eq. (52) expressed as follows [52]:

(52) $$T_{sky}=T_{amb}-20\;K$$

#### 2.2.7 Energy balance method

The proposed approach is based on the energy balance method, since it considers the energy entering, leaving, generated and stored inside the PV layers (see Eq. (53)).

(53) $${\dot{E}}_{ent}+{\dot{E}}_{g}={\dot{E}}_{st}+{\dot{E}}_{exit}$$

where the subscripts *ent*, *g*, *st* and *exit* are respectively, entering, generated, stored and exiting. These energies are expressed in rate form.

#### 2.2.8 The finite different method

The main purpose is to approximate the infinitesimal differences in the general heat equation using the Finite differences. This might lead to a series of equations and algebraic relations. Expanding the temperature terms using the Taylor series truncated at the second order, it is possible to approximate the derivatives of Eq. (45) into Eq. (54), (55), (56)

(54) $$\frac{\partial T\left(x,y,t\right)}{\partial t}=\frac{T\left(x,y,t+\Delta t\right)-T\left(x,y,t\right)}{\Delta t}=\frac{T_{Nx,Ny}^{p+1}-T_{Nx,Ny}^{p}}{\Delta t}$$

(55) $$\frac{\partial^{2}T\left(x,y,t\right)}{\partial^{2}x}=\frac{T_{Nx+1,Ny}-2T_{Nx,Ny}+T_{Nx-1,Ny}}{{\Delta x}^{2}}=\frac{T_{Nx+1,Ny}-2T_{Nx,Ny}+T_{Nx-1,Ny}}{\Delta x^{2}}$$

(56) $$\frac{\partial^{2}T\left(x,y,t\right)}{\partial^{2}y}=\frac{T_{Nx,Ny+1}-2T_{Nx,Ny}+T_{Nx,Ny-1}}{{\Delta y}^{2}}=\frac{T_{Nx,Ny+1}-2T_{Nx,Ny}+T_{Nx,Ny-1}}{\Delta y^{2}}$$

The choice of time step is done using the implicit time scheme, it is probably not as much accurate as the explicit time scheme, still it does not have too much constraints. The spatial derivatives of heat equations are solved at future time p+1 which is dependent of time p. Still, both values are unknown, which leads to the creation of a system of equations that need to be solved simultaneously to obtain the unknown temperature, at each node and each time step.

**Fig. 4:**
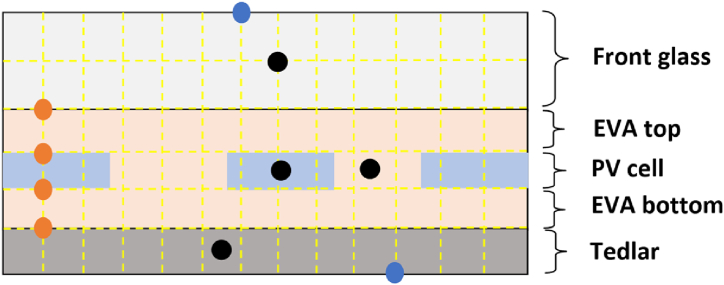
Schematic representation of the nodes in the PV layers: orange for interface nodes, black for internal nodes and blue for surface nodes. (For interpretation of the references to colour in this figure legend, the reader is referred to the Web version of this article.)

Fig. 4 below presents an example of the different node placement into/between the PV layers.

##### 2.2.8.1 Energy balance on the top/bottom surface nodes

**Fig. 5:**
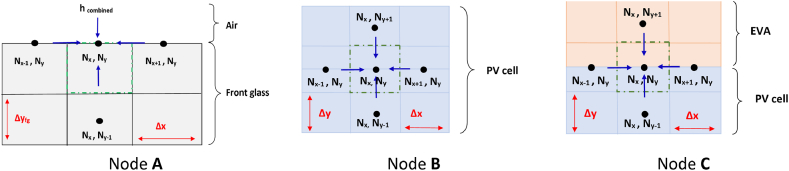
Schematic presentation of the nodes heat balances.

As can be seen from Fig. 3, both top and bottom surfaces exchange energy with the atmosphere (via convection and radiation) and the PV panel other layers (through conduction). Hence, the energy balance of these nodes will be considered as being similar (node A Fig. 5). Still, for concretization purposes, we take the example of node A, where the coordinate number of the node of interest is considered as (N_x_, N_y_). As for the neighboring nodes, the node (N_x_, N_y_) exchanges with the node (N_x+1_, N_y_), (N_x-1_, N_y_) and (N_x_, N_y-1_) through conduction and to the surroundings via radiation and convection. Applying energy balance, the elements ${\dot{E}}_{ent}$, ${\dot{E}}_{g}$, ${\dot{E}}_{st}$ will be expressed using, respectivel, Eqs. (57), (58), (59):

(57) $${\dot{E}}_{ent}=\lambda_{fg}\frac{\Delta y_{fg}}{2}\left(\frac{T_{N_{x-1},N_{y}}^{p+1}-T_{N_{x},N_{y}}^{p+1}}{\Delta x}\right)+\lambda_{fg}\frac{\Delta y_{fg}}{2}\left(\frac{T_{N_{x+1},N_{y}}^{p+1}-T_{N_{x},N_{y}}^{p+1}}{\Delta x}\right)+\lambda_{fg}\frac{\Delta x}{2}\left(\frac{T_{N_{x},N_{y-1}}^{p+1}-T_{N_{x},N_{y}}^{p+1}}{{\Delta y}_{fg}}\right)+h_{conv}\Delta x\left(T_{amb}^{p+1}-T_{N_{x},N_{y}}^{p+1}\right)+h_{rad,front}\Delta x\left(T_{sky}^{p+1}-T_{N_{x},N_{y}}^{p+1}\right)$$

(58) $${\dot{E}}_{g}=\Delta x\frac{{\Delta y}_{fg}}{2}Q_{fg}$$

(59) $${\dot{E}}_{st}=\left(\rho_{fg}C_{fg}\right)\left(\Delta x\frac{{\Delta y}_{fg}}{2}\right)\left(\frac{T_{N_{x},N_{y}}^{p+1}-T_{N_{x},N_{y}}^{p}}{\Delta t}\right)$$

Replacing each term with its expression, we get the general equation (60)

(60) $$\lambda_{fg}\frac{\Delta y_{fg}}{2}\left(\frac{T_{N_{x-1},N_{y}}^{p+1}-T_{N_{x},N_{y}}^{p+1}}{\Delta x}\right)+\lambda_{fg}\frac{\Delta y_{fg}}{2}\left(\frac{T_{N_{x+1},N_{y}}^{p+1}-T_{N_{x},N_{y}}^{p+1}}{\Delta x}\right)+\lambda_{fg}\frac{\Delta x}{2}\left(\frac{T_{N_{x},N_{y-1}}^{p+1}-T_{N_{x},N_{y}}^{p+1}}{{\Delta y}_{fg}}\right)+h_{conv}\Delta x\left(T_{amb}^{p+1}-T_{N_{x},N_{y}}^{p+1}\right)+h_{rad,front}\Delta x\left(T_{sky}^{p+1}-T_{N_{x},N_{y}}^{p+1}\right)+\Delta x\frac{{\Delta y}_{fg}}{2}Q_{fg}=\left(\rho_{fg}C_{fg}\right)\left(\Delta x\frac{{\Delta y}_{fg}}{2}\right)\left(\frac{T_{N_{x},N_{y}}^{p+1}-T_{N_{x},N_{y}}^{p}}{\Delta t}\right)$$

Hence, by rearranging the terms, we get the temperature of the specific control volume as expressed in Eq. (61) below:

(61) $$T_{N_{x},N_{y}}^{p}=\left(\frac{2\Delta t}{\rho_{fg}C_{fg}\Delta x{\Delta y}_{fg}}\right)\left[T_{N_{x},N_{y}}^{p+1}\left(\frac{\rho_{fg}C_{fg}\Delta x{\Delta y}_{fg}}{2\Delta t}+\frac{\lambda_{fg}{\Delta y}_{fg}}{\Delta x}+\frac{\lambda_{fg}\Delta x}{2{\Delta y}_{fg}}+h_{conv}\Delta x+h_{rad,front-sky}\Delta x+h_{rad,front-gr}\Delta x\right)-T_{N_{x-1},N_{y}}^{p+1}\left(\frac{\lambda_{fg}\Delta y_{fg}}{2\Delta x}\right)-T_{N_{x+1},N_{y}}^{p+1}\left(\frac{\lambda_{fg}\Delta y_{fg}}{2\Delta x}\right)-T_{N_{x},N_{y-1}}^{p+1}\left(\frac{\lambda_{fg}\Delta x}{2{\Delta y}_{fg}}\right)-T_{amb}^{p+1}\left(h_{conv}\Delta x\right)-T_{sky}^{p+1}\left(h_{rad,front-sky}\Delta x\right)-\Delta x\frac{{\Delta y}_{fg}}{2}Q_{fg}\right]$$

If we considered the node at the bottom surface of the panel, ${\dot{E}}_{g}$ would have been equal to zero since no heat generation is assumed in the Tedlar layer. $h_{rad,front}$ will be replaced by $h_{rad,back}$. However, the $h_{conv}$ is the same since both surfaces experience the same convective heat transfer conditions.

##### 2.2.8.2 Energy balance on interior nodes

When mentioning interior nodes, we mean the nodes that lay between the layers of the PV panels (nodes between EVA top and PV, between PV and EVA bottom and the node between EVA bottom and Tedlar). These nodes must be placed elsewhere than the edge so as not to be confused with the interface nodes (which will be discussed in the following section). For concretization purposed, we will present the equations related to node B from Fig. 5 (inside PV cell), where its coordinate number is considered as (N_x_, N_y_). This node exchanges heat with its surroundings; (N_x+1_, N_y_), (N_x-1_, N_y_), (N_x_, N_y-1_) and (N_x_, N_y+1_), via conduction only. Applying energy balance, the elements ${\dot{E}}_{ent}$, ${\dot{E}}_{g}$, ${\dot{E}}_{st}$ will be expressed using, respectivel, Eqs. (62), (63), (64):

(62) $${\dot{E}}_{ent}=\lambda_{PV}\Delta y\left(\frac{T_{N_{x-1},N_{y}}^{p+1}-T_{N_{x},N_{y}}^{p+1}}{\Delta x}\right)+\lambda_{PV}\Delta y\left(\frac{T_{N_{x+1},N_{y}}^{p+1}-T_{N_{x},N_{y}}^{p+1}}{\Delta x}\right)+\lambda_{PV}\Delta x\left(\frac{T_{N_{x},N_{y-1}}^{p+1}-T_{N_{x},N_{y}}^{p+1}}{\Delta y}\right)+\lambda_{PV}\Delta x\left(\frac{T_{N_{x},N_{y+1}}^{p+1}-T_{N_{x},N_{y}}^{p+1}}{\Delta y}\right)$$

(63) $${\dot{E}}_{g}=\Delta x\Delta yQ_{PV}$$

(64) $${\dot{E}}_{st}=\left(\rho_{PV}C_{PV}\right)\left(\Delta x\Delta y\right)\left(\frac{T_{N_{x},N_{y}}^{p+1}-T_{N_{x},N_{y}}^{p}}{\Delta t}\right)$$

Replacing each term with its expression, we get the general equation (65)

(65) $$\lambda_{PV}\Delta y\left(\frac{T_{N_{x-1},N_{y}}^{p+1}-T_{N_{x},N_{y}}^{p+1}}{\Delta x}\right)+\lambda_{PV}\Delta y\left(\frac{T_{N_{x+1},N_{y}}^{p+1}-T_{N_{x},N_{y}}^{p+1}}{\Delta x}\right)+\lambda_{PV}\Delta x\left(\frac{T_{N_{x},N_{y-1}}^{p+1}-T_{N_{x},N_{y}}^{p+1}}{\Delta y}\right)+\lambda_{PV}\Delta x\left(\frac{T_{N_{x},N_{y+1}}^{p+1}-T_{N_{x},N_{y}}^{p+1}}{\Delta y}\right)+\Delta x\Delta yQ_{PV}=\left(\rho_{PV}C_{PV}\right)\left(\Delta x\Delta y\right)\left(\frac{T_{N_{x},N_{y}}^{p+1}-T_{N_{x},N_{y}}^{p}}{\Delta t}\right)$$

Hence, by rearranging the terms, we get the temperature of the specific control volume as expressed in Eq. (66)

(66) $$T_{N_{x},N_{y}}^{p}=\frac{\Delta t}{\rho_{PV}C_{PV}\Delta x\Delta y}\left[-T_{N_{x},N_{y}}^{p+1}\left(2\frac{\lambda_{PV}\Delta y}{\Delta x}+2\frac{\lambda_{PV}\Delta x}{\Delta y}+\frac{\rho_{PV}C_{PV}\Delta x\Delta y}{\Delta t}\right)+\frac{\lambda_{PV}\Delta y}{\Delta x}T_{N_{x-1},N_{y}}^{p+1}+\frac{\lambda_{PV}\Delta y}{\Delta x}T_{N_{x+1},N_{y}}^{p+1}+\frac{\lambda_{PV}\Delta x}{\Delta y}T_{N_{x},N_{y-1}}^{p+1}+\frac{\lambda_{PV}\Delta x}{\Delta y}T_{N_{x},N_{y+1}}^{p+1}+\Delta x\Delta yQ_{PV}\right]$$

If the node belongs to some other layer than the PV layers, then the thermal characteristics will vary accordingly. If the node belongs to the front glass, then $Q_{PV}$ will be replaced by $Q_{fg}$, if not then zero (since no other layer than these two generates heat).

##### 2.2.8.3 Energy balance on the interface nodes

Nodes placed at the edges of two layers are called interface nodes. In the concerned case of study, we find four interface nodes: front glass/EVA top, EVA top/PV cell, PV cell/EVA bottom, EVA bottom/Tedlar. For concretization purposes, we will consider the case of the interface between EVA top and PV cell (Node C from Fig. 4). Applying energy balance, the elements ${\dot{E}}_{ent}$, ${\dot{E}}_{g}$, ${\dot{E}}_{st}$ will be expressed using, respectivel, Eqs. (67), (68), (69):

(67) $${\dot{E}}_{ent}=\lambda_{EVA\;top}\Delta x\left(\frac{T_{N_{x},N_{y+1}}^{p+1}-T_{N_{x},N_{y}}^{p+1}}{\Delta y}\right)+\lambda_{PV}\Delta x\left(\frac{T_{N_{x},N_{y-1}}^{p+1}-T_{N_{x},N_{y}}^{p+1}}{\Delta y}\right)+\left(\lambda_{PV}+\lambda_{EVA\;top}\right)\frac{\Delta y}{2}\left(\frac{T_{N_{x-1},N_{y}}^{p+1}-T_{N_{x},N_{y}}^{p+1}}{\Delta x}\right)+\left(\lambda_{PV}+\lambda_{EVA\;top}\;\right)\frac{\Delta y}{2}\left(\frac{T_{N_{x+1},N_{y}}^{p+1}-T_{N_{x},N_{y}}^{p+1}}{\Delta x}\right)$$

(68) $${\dot{E}}_{g}=\Delta x\frac{\Delta y}{2}Q_{PV}$$

(69) $${\dot{E}}_{st}=\left(\rho_{EVA}C_{EVA\;top}+\rho_{PV}C_{PV}\right)\left(\Delta x\frac{\Delta y}{2}\right)\left(\frac{T_{N_{x},N_{y}}^{p+1}-T_{N_{x},N_{y}}^{p}}{\Delta t}\right)$$

Hence, by rearranging the terms, we get the temperature of the specific control volume as expressed in Eq. (70)

(70) $$T_{N_{x},N_{y}}^{p}=\left(\frac{2\Delta t}{\left(\rho_{EVA}C_{EVA\;top}+\rho_{PV}C_{PV}\right)\;\Delta x\Delta y}\right)\left[T_{N_{x},N_{y}}^{p+1}\left(\frac{\left(\rho_{EVA}C_{EVA\;top}+\rho_{PV}C_{PV}\right)\;\Delta x\Delta y}{2\Delta t}+\frac{\lambda_{PV}\Delta x}{\Delta y}+\frac{\lambda_{EVA\;top}\Delta x}{\Delta y}+\frac{\left(\lambda_{EVA\;top}+\lambda_{PV}\right)\;\Delta y}{\Delta x}\right)-T_{N_{x-1},N_{y}}^{p+1}\left(\frac{\left(\lambda_{EVA\;top}+\lambda_{PV}\right)\;\Delta y}{2\Delta x}\right)-T_{N_{x+1},N_{y}}^{p+1}\left(\frac{\left(\lambda_{EVA\;top}+\lambda_{PV}\right)\;\Delta y}{2\Delta x}\right)-T_{N_{x},N_{y-1}}^{p+1}\left(\frac{\lambda_{EVA\;top}\Delta x}{\Delta y}\right)-T_{N_{x},N_{y-1}}^{p+1}\left(\frac{\lambda_{PV}\Delta x}{\Delta y}\right)-\left(\Delta x\frac{\Delta y}{2}\right)Q_{PV}\right]$$

For all forementioned equations (57), (70), λ is the conductivity of the material, ρ is the density, C is the thermal specific heat capacity, (x,y) are space coordinates, and t is the time, Δx is the length of control in the x-axis direction, Δy is the length of control in the y-axis direction, T is temperature of each node (Nx, Ny), Q is internal heat generation and can be found throughout Eqs. (46), (47). The subscripts *fg*, *EVA*, *PV* and *Tdl*, respectively, refer to front glass, EVA, PV cell and Tedlar back sheet.

As can be noticed, there is a slight difference among node A and the rest in terms of spatial distribution among the y-axis, ${\Delta y}_{fg}$ was set for glass layer as for the rest, they were all set to Δy.

As was previously mentioned, adopting the implicit time scheme let us with various unknown parameters (mainly, temperature at time p+1 at different nodes) which necessitates all equations being simultaneously solved. This can be easily done through the rearrangement of all equations in matrix form as presented belo in Eq. (71)

(71) [A]{T}={C}

Where [A] is a sparse tridiagonal square matrix of size N x N and it englobes the elements from terms adjacent to temperature. {T} is the vector including the unknown nodal temperature. Vector {C} has constants involving quantities and known temperatures at time p. Eq. (72) can be further expressed as shown in Eq. (72)

(72) $$\left[\begin{array}{ccc} \begin{array}{cc} A_{1,1} & A_{1,2}\\ A_{2,1} & A_{2,2} \end{array} & \cdots & \begin{array}{cc} A_{1,n-1} & A_{1,n}\\ A_{2,n-1} & A_{2,n} \end{array}\\ \vdots & \ddots & \vdots \\ \begin{array}{cc} A_{n-1,1} & A_{n-1,2}\\ A_{n,1} & A_{n,2} \end{array} & \cdots & \begin{array}{cc} A_{n-1,n-1} & A_{n-1,n}\\ A_{n,n-1} & A_{n,n} \end{array} \end{array}\right]\times \left\{\begin{array}{c} T_{1}^{p+1}\\ T_{2}^{p+1}\\ \begin{array}{c} \vdots \\ T_{n-1}^{p+1}\\ T_{n}^{p+1} \end{array} \end{array}\right\}=\left\{\begin{array}{c} C_{1}\\ C_{2}\\ \begin{array}{c} \vdots \\ C_{n-1}\\ C_{n} \end{array} \end{array}\right\}$$

In order to resolve the equation and obtain temperature distribution, it is possible through the inversion of the matrix as shown in Eq. (73) below:

(73) {T}=inv[A]×{C}

#### 2.2.9 Elements of A and C

##### 2.2.9.1 From top/bottom surface nodes

Similar to what has been presented in section 2.2.8.1, we present the case of the front glass surface, where its corresponding nodal expression is given in Eq. (61). From this latter, we will present how to extract the elements of the matrix A and C. As can be noticed in Fig. 5, the front glass has three adjacent nodes, (Nx, Ny-1), (Nx-1, Ny) and (Nx+1, Ny). Which means that this particular node will give four entries of matrix A, one from the node itself (i.e., coefficient next to $T_{N_{x},N_{y}}^{p+1}$), and three from the adjacent nodes (i.e., coefficient next to $T_{N_{x-1},N_{y}}^{,p+1},T_{N_{x+1},N_{y}}^{p+1}\;and\;T_{N,N_{y-1}}^{p+1}$). As for the elements of vector C, it represents the sum of all terms in equation (61) which are node related to any temperature term in addition to $T_{N_{x},N_{y}}^{p}$ term. The elements of matrix A and C are presented in Table 4, where a similar procedure shall be used to extract elements of the bottom surface (the one of the Tedlar layer).

**Table 4:** Elements of matrix A and B from front glass surface nodes.

$A\left(N_{x},N_{y}\right)$	$\left(\frac{2\Delta t}{\rho_{fg}C_{fg}\Delta x{\Delta y}_{fg}}\right)\left(\frac{\rho_{fg}C_{fg}\Delta x{\Delta y}_{fg}}{2\Delta t}+\frac{\lambda_{fg}{\Delta y}_{fg}}{\Delta x}+\frac{\lambda_{fg}\Delta x}{2{\Delta y}_{fg}}+h_{conv}\Delta x+h_{rad,front-sky}\Delta x\right)$
$A\left(N_{x-1},N_{y}\right)$	$-\left(\frac{2\Delta t}{\rho_{fg}C_{fg}\Delta x{\Delta y}_{fg}}\right)\left(\frac{\lambda_{fg}\Delta y_{fg}}{2\Delta x}\right)$
$A\left(N_{x+1},N_{y}\right)$	$-\left(\frac{2\Delta t}{\rho_{fg}C_{fg}\Delta x{\Delta y}_{fg}}\right)\left(\frac{\lambda_{fg}\Delta y_{fg}}{2\Delta x}\right)$
$A\left(N_{x},N_{y-1}\right)$	$-\left(\frac{2\Delta t}{\rho_{fg}C_{fg}\Delta x{\Delta y}_{fg}}\right)\left(\frac{\lambda_{fg}\Delta x}{2{\Delta y}_{fg}}\right)$
$C\left(N_{x},N_{y}\right)$	$+\left(\frac{2\Delta t}{\rho_{fg}C_{fg}\Delta x{\Delta y}_{fg}}\right)\left(T_{amb}^{p+1}\left(h_{conv}\Delta x\right)+T_{sky}^{p+1}\left(h_{rad,front-sky}\Delta x\right)+\Delta x\frac{{\Delta y}_{fg}}{2}Q_{fg}\right)+T_{N_{x},N_{y}}^{p}$

##### 2.2.9.2 From interior nodes

Similar to what has been presented in section 2.2.8.2, we present the case of the PV cell layer, where its corresponding nodal expression is given in Eq. (66). From this latter, we will present how to extract the elements of the matrix A and C. As can be noticed in Fig. 4, the PV cell has four adjacent nodes, (Nx, Ny-1), (Nx-1, Ny), (Nx+1, Ny) and (Nx, Ny+1). Which means that this particular node will give five entries of matrix A, one from the node itself (i.e., coefficient next to $T_{N_{x},N_{y}}^{p+1}$), and three from the adjacent nodes (i.e., coefficient next to $T_{N_{x-1},N_{y}}^{,p+1},T_{N_{x+1},N_{y}}^{p+1},T_{N,N_{y-1}}^{p+1}and\;T_{N,N_{y+1}}^{p+1}$). Similarly, the elements of vector C represent the sum of all terms in Eq. (66) which are node related to any temperature term in addition to $T_{N_{x},N_{y}}^{p}$ term. The elements of matrix A and C are presented in Table 5, where a similar procedure shall be used to extract elements from other internal nodes (for different layers).

**Table 5:** Elements of matrix A and B from interior surface nodes: PV cell.

$A\left(N_{x},N_{y}\right)$	$+\left(\frac{\Delta t}{\rho_{PV}C_{PV}\Delta x\Delta y}\right)\left(2\frac{\lambda_{PV}\Delta y}{\Delta x}+2\frac{\lambda_{PV}\Delta x}{\Delta y}+\frac{\rho_{PV}C_{PV}\Delta x\Delta y}{\Delta t}\right)$
$A\left(N_{x-1},N_{y}\right)$	$-\left(\frac{\Delta t}{\rho_{PV}C_{PV}\Delta x\Delta y}\right)\left(\frac{\lambda_{PV}\Delta y}{\Delta x}\right)$
$A\left(N_{x+1},N_{y}\right)$	$-\left(\frac{\Delta t}{\rho_{PV}C_{PV}\Delta x\Delta y}\right)\left(\frac{\lambda_{PV}\Delta y}{\Delta x}\right)$
$A\left(N_{x},N_{y+1}\right)$	$-\left(\frac{\Delta t}{\rho_{PV}C_{PV}\Delta x\Delta y}\right)\left(\frac{\lambda_{PV}\Delta x}{\Delta y}\right)$
$A\left(N_{x},N_{y-1}\right)$	$-\left(\frac{\Delta t}{\rho_{PV}C_{PV}\Delta x\Delta y}\right)\left(\frac{\lambda_{PV}\Delta x}{\Delta y}\right)$
$C\left(N_{x},N_{y}\right)$	$\frac{\Delta t}{\rho_{PV}C_{PV}\Delta x\Delta y}\left(\Delta x\Delta yQ_{PV}\right)+T_{N_{x},N_{y}}^{p}$

##### 2.2.9.3 From interior nodes

Similar to what has been presented in section 2.2.8.3, we present the case of the interface between the PV cell layer and the EVA top, where its corresponding nodal expression is given in Eq. (70). From this latter, we will present how to extract the elements of the matrix A and C. As can be noticed in Fig. 5, the PV cell has four adjacent nodes, (Nx, Ny-1), (Nx-1, Ny), (Nx+1, Ny) and (Nx, Ny+1). Which means that this particular node will give five entries of matrix A, one from the node itself (i.e., coefficient next to $T_{N_{x},N_{y}}^{p+1}$), and three from the adjacent nodes (i.e., coefficient next to $T_{N_{x-1},N_{y}}^{,p+1},T_{N_{x+1},N_{y}}^{p+1},T_{N,N_{y-1}}^{p+1}and\;T_{N,N_{y+1}}^{p+1}$). Similarly, the elements of vector C represent the sum of all terms in Eq. (70) which are node related to any temperature term in addition to $T_{N_{x},N_{y}}^{p}$ term. The elements of matrix A and C are presented in Table 6, where a similar procedure shall be used to extract elements from other interface nodes.

**Table 6:** Elements of matrix A and B from interface nodes: PV cell and EVA top.

$A\left(N_{x},N_{y}\right)$	$\left(\frac{2\Delta t}{\left(\rho_{EVA-Top}C_{EVA-Top}+\rho_{\text{PV}}C_{\text{PV}}\right)\;\Delta x\Delta y}\right)\left(\frac{\left(\rho_{EVA-Top}C_{EVA-Top}+\rho_{\text{PV}}C_{\text{PV}}\right)\;\Delta x\Delta y}{2\Delta t}+\frac{\lambda_{\text{PV}}\Delta x}{\Delta y}+\frac{\lambda_{EVA-Top}\Delta x}{\Delta y}+\frac{\left(\lambda_{EVA-Top}+\lambda_{\text{PV}}\right)\;\Delta y}{\Delta x}\right)$
$A\left(N_{x-1},N_{y}\right)$	$-\left(\frac{2\Delta t}{\left(\rho_{\text{EVA}-\text{Top}}C_{\text{EVA}-\text{Top}}+\rho_{\text{PV}}C_{\text{PV}}\right)\;\Delta x\Delta y}\right)\left(\frac{\left(\lambda_{\text{EVA}-\text{Top}}+\lambda_{\text{PV}}\right)\;\Delta y}{2\Delta x}\right)$
$A\left(N_{x+1},N_{y}\right)$	$-\left(\frac{2\Delta t}{\left(\rho_{\text{EVA}-\text{Top}}C_{\text{EVA}-\text{Top}}+\rho_{\text{PV}}C_{\text{PV}}\right)\;\Delta x\Delta y}\right)\left(\frac{\left(\lambda_{\text{EVA}-\text{Top}}+\lambda_{\text{PV}}\right)\;\Delta y}{2\Delta x}\right)$
$A\left(N_{x},N_{y+1}\right)$	$-\left(\frac{2\Delta t}{\left(\rho_{\text{EVA}-\text{Top}}C_{\text{EVA}-\text{Top}}+\rho_{\text{PV}}C_{\text{PV}}\right)\;\Delta x\Delta y}\right)\left(\frac{\lambda_{\text{EVA}-\text{Top}}\Delta x}{\Delta y}\right)$
$A\left(N_{x},N_{y-1}\right)$	$-\left(\frac{2\Delta t}{\left(\rho_{\text{EVA}-\text{Top}}C_{\text{EVA}-\text{Top}}+\rho_{\text{PV}}C_{\text{PV}}\right)\;\Delta x\Delta y}\right)\left(\frac{\lambda_{\text{PV}}\Delta x}{\Delta y}\right)$
$C\left(N_{x},N_{y}\right)$	${+\left(\frac{2\Delta t}{\left(\rho_{\text{EVA}-\text{Top}}C_{\text{EVA}-\text{Top}}+\rho_{\text{PV}}C_{\text{PV}}\right)\;\Delta x\Delta y}\right)\left(\Delta x\frac{\Delta y}{2}Q_{\text{PV}}\right)+T}_{N_{x},N_{y}}^{p}$

#### 2.2.10 Implementation procedure

Once the analytical model has been developed, it has been implemented into the python program as to assess PV temperature values according to varying weather conditions and to the linked electrical charges. The implementation procedure is described as follows:

%9. Import field data, such as wind speed, irradiances, Tgr and Tamb.

%9. Describe constants (β, $\theta_{z}$ , g ,F, ….etc)

%9. Define the step size (t) and grid size (m, n).

%9. List the dimensions and material characteristics for each layer.

%9. Initialize the vectors and matrices (A, T and C)

%9. While t is smaller than the final simulation time, or the solution has not converged (only for steady state)

%9. Determine variables, such as T_amb_, T_sky_, air characteristics, etc.

%9. Dial the radiation model to determine the absorbed radiation (S)

%9. Determine parameters depending on S, such as Q_pv_, Q_fg_,

%9. Resolve equation T = inv([A]){C}

%9. Keep essential variables (such as T_PV_,T__fg_ and T__Td_ etc.) in each cycle.

%9. Replace Tp with Tp+1, then continue the loop with t + Δt in its place.

#### 2.2.11 Coupling

**Fig. 6:**
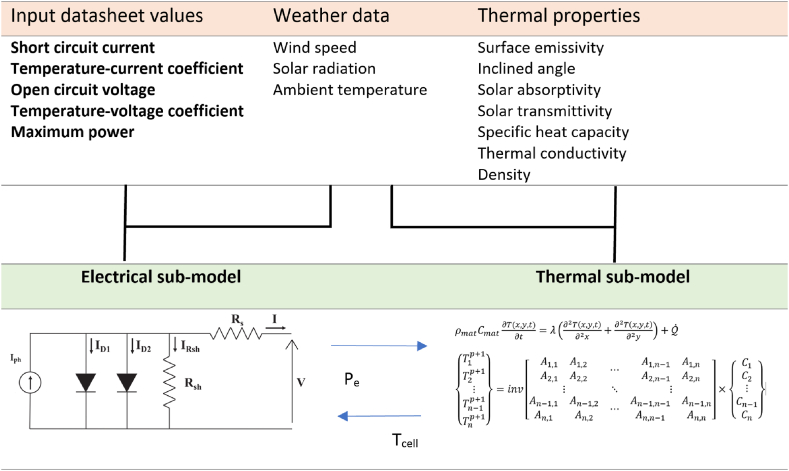
Scheme of the sub-models coupling.

Knowing the temperature of the solar cell is important to have an accurate estimation of the panel's electrical performance. Simultaneously, it is important to develop a thermal model that considers the electrical performance as to have accurate temperature distribution. In this sub-section, a schematic representation (see Fig. 6) of the coupling of both models is presented.

### 2.3 Experimental setup

**Fig. 7:**
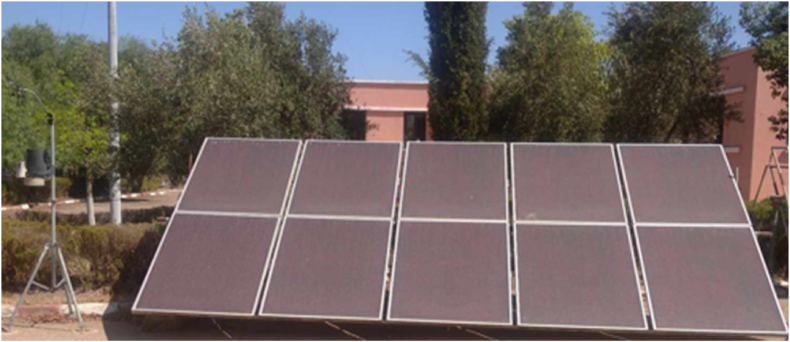
Deployed scenario of the thin-film modules.

In this section, a description of the overall experimental setup that has been adopted by this study is presented. The systems are installed at the National Institute for Agronomic Research (INRA) located in Settat, Morocco. Two main PV technologies are installed in the site, namely, Monocrystalline and Thin-film layer PV systems. Still, in this study, focus has been directed towards the thin-film layer PV system. The setup is a 100W Thin-Film MITSUBISHI solar PV with Uoc = 141V and Isc = 1.17A. Fig. 7 show the deployed scenario.

The setup is used for research and development purposes at INRA and it includes a PV generator, DC/DC converter and a variable electric load to simulate different electric loads and study their effect on the upstream thermal behavior.

In terms of DC/DC converter, the Victron blue solar 48/24/12V solar inverter is used to regulate the input voltage feeding the load to optimize the delivered power. As for the load variation, three different DC lamps have been used verifying at 22W, 35W and 55W.

**Fig. 8:**
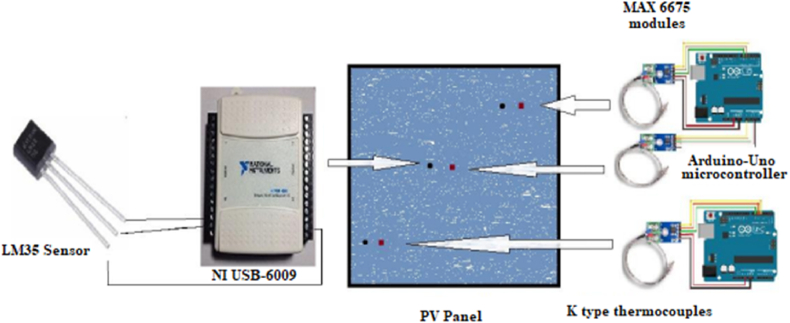
Schematic representation of the sensors placed at the PV panel.

The temperatures of the PV panels have been collected using K type thermocouple coupled to MAX 6675 modules, connected to an Arduino Uno microcontroller, and mounted at the back of the PV panels to avoid readings errors generated from the direct solar radiation hitting the sensors (see Fig. 8). Moreover, LM35 coupled to DAQ USB NI-6009 data acquisition device have been used to provide readings of temperatures. However, to read the PV panels’ surface temperatures, a Fluke multimeter with temperature probe has been used. Furthermore, a thermal infrared camera of type Flir E60bx is used to capture thermal images of the installed panels.

Two series connected current sensors ACS712 have been used before and after the solar inverter to collect its input and output currents. Regarding the weather data, the wireless Vantage Pro2 weather station has been used to provide data of various sensors. This type of station is available in both wired and wireless versions, which avoids the constraints of retrieving data from difficult-to-access installations. For wired configuration, the console is installed on site, with its data logger connected to the model. Data is exchanged using WeatherLink software and a PSTN modem, with a distance of 30 m between console and sensors, but can be extended up to 300 m if needed.

The PV panels are connected in parallel to the input of the inverter and controlled using switchers to activate or deactivate the panel, while the output is connected to a 22W lamp, then 35W and 55W, and finally two 55W lamps for 110W. The thermal and electrical behavior of the setup are monitored at various hours and under different daytime illumination. Fig. 8 shows a schematic representation of the setup in which sensors are mounted on the back of the PV panel.

Both Table 7, Table 8 presents the experimental instruments used for this study and their specifications.

**Table 7:** The key experimental instruments and their specifications.

Experiment equipment	Model	Specification	Measurement error
Solar Pyranometer	TBQ-2	Sensitivity: 7–14 μV/(W∙m-2) Output signal 0–20 mV	<5%
Thermocouples	PT 100	Temperature range −50 to 200 °C	±0.2 °C
Data logger	DAM3232 data acquisition controller	Power supply voltage: DC 12/24 V Temperature range: 40 to 85 °C	–

**Table 8:** Sensors’ specifications.

	Temperature	Relative humidity	Wind direction	Wind speed
Sensor	Pt 100 1/3	Capacitive	No contact hall	
Range	−50 … 70 (°C)	0 … 100 (%)	0 … 360 (°C)	0 … 75 (m/s)
Accuracy	0.1 (0 °C)	±1.5 (RH5-95%)	5 (°)	2.5 %

As for the use of the infrared camera, at each experimental setup and under the different scenarios, thermal images are captured to first measure the temperature and observe its distribution on the surface of the PV panels.

## 3 Results and discussion

### 3.1 Validation of the proposed models

The proposed models; mainly the electrical and the coupled; have been tested for accuracy using two different experimental data. The electrical model was first independently validated; using results issued from experimentation [53]; before being coupled with the thermal model to evaluate how well the developed finite difference thermal model performed in estimating the temperature of the PV panel cell. The overall coupled model has been validated using the experimental data described in section 2.3.

#### 3.1.1 Electrical model results

**Fig. 9:**
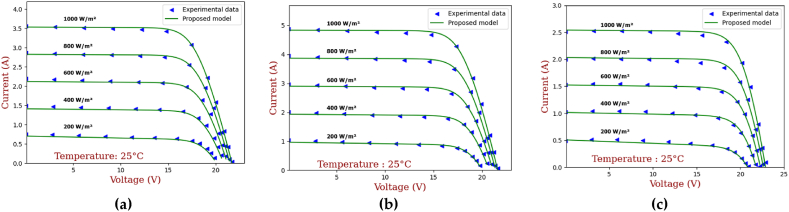
IV curves for different irradiation levels (a) SM55 (monocrystalline), (b) S75 (multi-crystalline) and (c) ST36 (thin film).

**Fig. 10:**
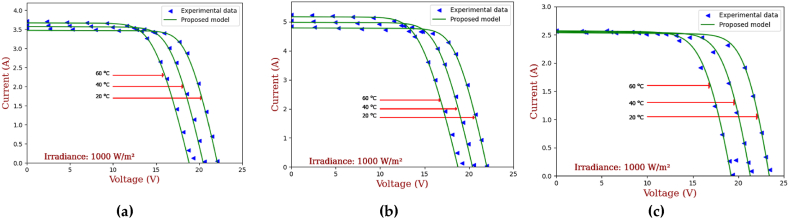
The IV curves for different temperature levels (a) SM55 (monocrystalline), (b) S75 (multi-crystalline) and (c) ST36 (thin film).

To validate the proposed electrical model, experimental data from Ref. [53] were used to generate characteristic curves for different types of photovoltaic (PV) panels, including SM55 mono-crystalline and S75 poly-crystalline and ST36 Thin-film. Fig. 9, Fig. 10, illustrate the impact of solar radiation and temperature on characteristic curves for the three types of PV panels at a cell temperature of 25 °C. For a fixed temperature of 25 °C, and as can be seen from Fig. 9, as the irradiance increases, the voltage experiences a minor, almost imperceptible increase, while the current output rises significantly.

Concerning Monocrystalline panels (see Fig. 9. (a)), they have a generally linear response to varying irradiance values. In fact, high irradiances lead to high current outputs, hence the I–V curve shifts upwards along the current axis while maintaining a consistent shape. Similarly, multicrystalline panels (see Fig. 9. (b)) show an increase in current while the irradiance increases. The I–V curve then shifts to imitate the same behavior as the monocrystalline panels. Still, small changes are noticed due to the difference between the materials. Thin film panels have a nonlinear response to changes in irradiance (see Fig. 9. (c)). A low irradiance values, they might exhibit a higher voltage and lower current compared to the monocrystalline panels.

Fig. 10 demonstrates the I–V curve behavior for different types of PV panels. In fact, the solar radiation has been maintained at 1000 W/m^2^, as for temperatures, they have been varied from 20 °C to 40 °C then 60 °C. In general, a rise in temperature leads to a marginal increase in output current while causing a substantial decrease in output voltage. This phenomenon ultimately results in a reduction in the overall efficiency of the panel. Furthermore, the effect is more pronounced with S75 panels (see Fig. 10 (b)), thanks to their higher temperature coefficient. These panels exhibit a more significant increase in current output as the temperature rises. Interestingly, thin film panels (see Fig. 10. (c)) appear to be less susceptible to the influence of temperature increases, maintaining a relatively stable current output throughout the entire temperature range in comparison to monocrystalline (Fig. 10 (a)) and multi-crystalline (Fig. 10 (b)).

The results indicate that the proposed electrical model aligns closely with the measured data, regardless of the PV technology used. Although minor deviations were observed around the maximum power point (MPP) under higher temperature, these discrepancies were less than 2%, which is deemed acceptable and consistent with ofther research. Consequently, the findings demonstrate that the electrical model developed in section 2 is a reliable tool for accurately estimating the electrical performance of a PV module. The high performance of the model can be attributed to the usage of the AHA and the accurate choice of values of its related parameters (number of iterations, population, etc.), allowing the model to converge to satisfactory solutions within a different range of constraints.

#### 3.1.2 Coupled thermal model results

**Fig. 11:**
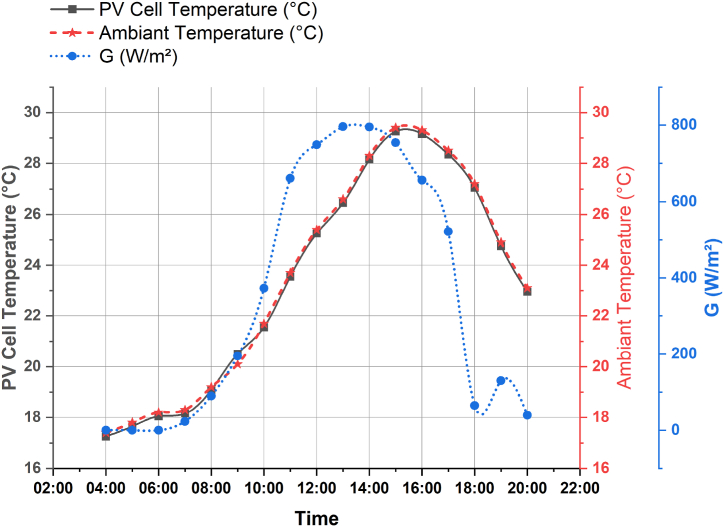
PV temperature in function of weather data variation.

To validate the results issued from the coupling procedure of both electrical and thermal models, the experimental setup explained in section 2.3 has been deployed. Fig. 11 presents the evolution of PV temperatures in function of time and weather data (from 4am till 8pm). For more accurate results and for a better understanding of the phenomenon, we focused our interest on 4 different hours to track front glass and tedlar's temperature evolution.

**Fig. 12:**
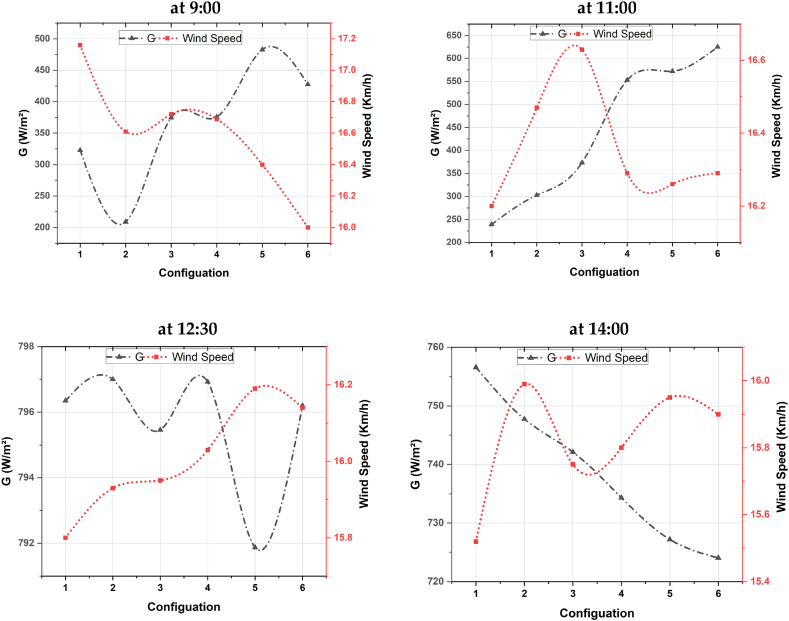
Irradiation and wind speed evolution at different hours (configurations).

Fig. 12 of both solar radiation and wind speed at different hours (at 9, 11, 12:30 and 14 h) of the July 6, 2021, and while applying different configurations (free load, 22W, 35W, 55W and 110W). It is to note that these configurations (at this level) do not interfere with the results of weather data. It is noticed that radiation attains it maximum at 12:30 to reach about 798 W, as for the wind speed, it reaches its maximum (17 Km/h) at early hours of the day (before 9:00). Defining peak hours is helpful for understanding the behavior of PV panels at when is supposed to be their best performance time.

**Fig. 13:**
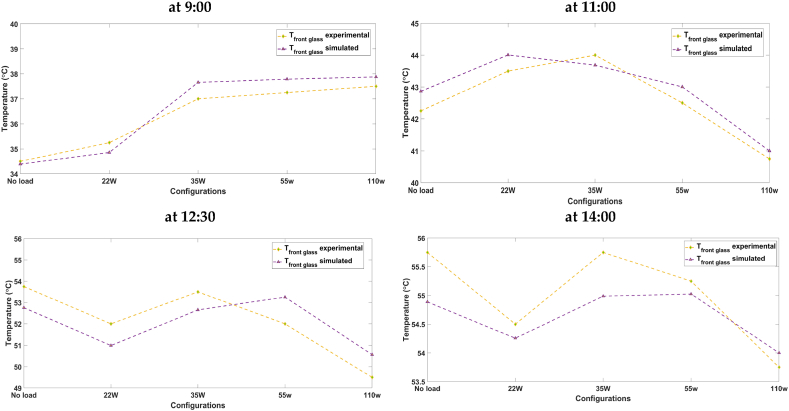
Front glass's experimental and simulated temperature, in response to various loads, under different weather conditions.

Fig. 13 shows the temperature values at the surface of the front glass, between simulation and experimentation, at different hours of the day and in response to varying loads. It is noticed that the curves have almost the same qualitative aspect, meaning that the temperature evolution in response to different loads have strong correlation between experimentation and simulation. The results show a good agreement between experimentation and simulation since the maximum deviation does not exceed 1 °C.

**Fig. 14:**
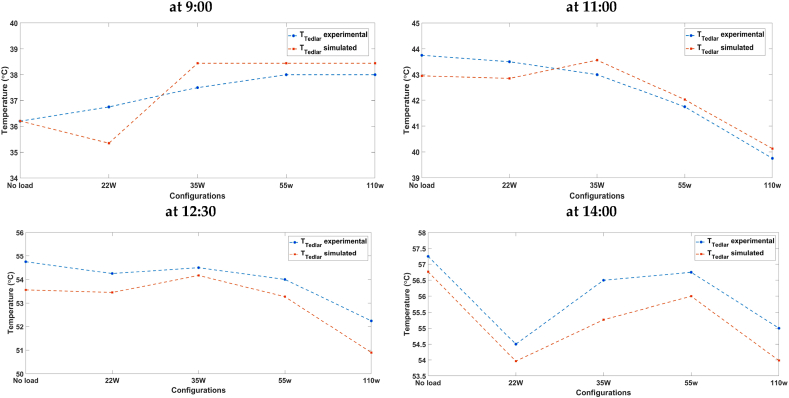
Tedlar's experimental and simulated temperature, in response to various loads, under different weather conditions.

Fig. 14 presents Tedlar's temperature in response to dynamic meteorological data. It is generally noticed that the difference between simulation and experimentation doesn't surpass a maximum of 1.5 °C. At 9 and 11 a.m., both graphs don't have the same qualitative behavior until loads superior than 35W are used. As for the other 2 h, both graphs have the same evolution aspect regardless of the power of the charge connected to the PV.

From Fig. 13, Fig. 14, we can notice that surface temperatures, especially the front glass; increases with the increase of ambient temperature and the linked load power. This is also correct for the back surface (Tedlar), still the effect of shadow attenuates the phenomenon.

**Fig. 15:**
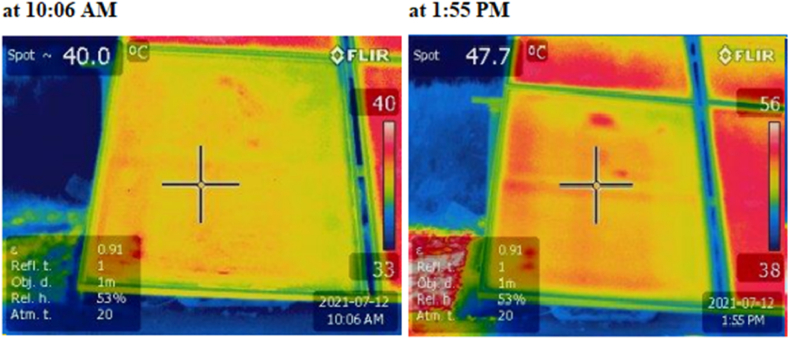
Front glass temperature evolution using infrared thermography.

To back up this observation, Fig. 15 presents panels temperature distribution in response to two different solar radiation values (at 10:00 and 13:55), using an infrared camera. As can be seen, the temperature of the front glass at 10:06 is at the range of 40 °C, whilst it increases at 13:55 to reach 47.7 °C since the ambient temperature and solar radiation increased as well.

Focusing on temperature distribution among the surface of the panel at each period independently, it can be noticed that at 10 a.m., temperature distribution is globally uniform. However, at 13:55 some hotspots seem to be appearing, this can be the index of some problems such as damaged areas or malfunctioning areas. This is mainly the result of problems such as short-circuited cells, dirt accumulation or other electrical issues.

## 4 Conclusions

In this present work, a novel model combining the electrical and thermal sub-models of PV performance assessment is presented. The electrical sub-model is based on a double diode configuration with accurate parameter extraction using the AHA algorithm. As for the thermal sub-model, it consists of a two-dimensional finite difference model that accounts for all thermal transfer phenomena as to assess the panel's temperature distribution. The models have been implemented using Python. Results of simulation have been compared to those of experimentation and showed a very good agreement, since the error for all models does not exceed 2%. The model proved its accuracy and its potential at being used for improvement of the research and development of PV technologies.

## Data availability statement

Data will be made available upon request.

## CRediT authorship contribution statement

**Radouane Aalloul:** Writing – original draft, Software, Project administration, Methodology, Investigation, Conceptualization. **Abdellah Elaissaoui:** Writing – review & editing, Validation, Supervision, Resources, Project administration, Methodology, Formal analysis, Conceptualization. **Assia Harkani:** Visualization, Validation, Resources, Formal analysis, Data curation. **Rhma Adhiri:** Writing – review & editing, Validation, Supervision, Methodology. **Mourad Benlattar:** Writing – review & editing, Validation, Supervision, Methodology, Investigation.

## Declaration of competing interest

The authors declare that they have no known competing financial interests or personal relationships that could have appeared to influence the work reported in this paper.

## Appendix A

1. Convective heat exchange

The convective heat exchange shall consider both the natural and forced contributions. The convective heat losses can be expressed as follows:

(74) $$q_{conv}=h_{conv}\left(T_{surf}-T_{amb}\right)$$

where the subscript *surf* can either be the front glass or the Tedlar.

a. Natural convection

Natural heat exchange coefficient can be calculated using some classical correlations based on the calculation of the Nusselt number, the Prandtl and Grashof numbers.

(75) $$h_{conv,nat}=\frac{Nu∙\lambda_{air}}{L}$$

The Nusselt number in Eq. (75) can be assumed as follows:

$$Nu=0.76∙{Ra}^{1/4}\;\text{for}\;10^{4}<Ra<10^{7}$$

$$Nu=0.15∙{Ra}^{1/3}\;\text{for}\;10^{7}<Ra<{3∙10}^{10}$$

Where Ra represents the Rayleigh number, dependent on both the Grashof (Gr) and the Prandtl (Pr) numbers:

(76) Ra=Gr.Pr

(77) $$Gr=\frac{g∙\beta_{air}∙\left(T_{PV,surf}-T_{amb}\right)∙L^{3}}{\nu^{2}}$$

(78) $$\mathrm{Pr}=\frac{C_{air}∙\nu ∙\rho_{air}}{\lambda_{air}}$$

where g is the universal gravitation, $\beta_{air}$ is the thermal expansion, $T_{PV,surf}$ is the surface temperature of the panel, $T_{amb}$ is the ambient temperature, L is the characteristic length of the PV panel, ν is the kinetic viscosity, $C_{air}$ is the thermal capacity of air, $\rho_{air}$ is the air density and $\lambda_{air}$ is the thermal conductivity of air.

The characteristic length can be determined by:

(79) $$L=4∙\frac{S_{PV}}{P_{PV}}$$

where, $S_{PV}$ is the area of the PV while $P_{PV}$ is the perimeter of the panel.

b. Forced convection

Since the heat transfer models are the ones presenting most uncertainties, in this presented work, it has been decided to adopt three different formulas as to evaluate the forced convective heat transfer coefficient.

• The first approach considers that the heat transfer coefficient is dependent on wind speed, as expressed in Eq. (80)

(80) $$h_{conv,for}=3∙V_{wind}+2.8$$

where $V_{wind}$ is the wind speed in (m/s)

• The second approach links the heat transfer coefficient to the regime of the flow.

for Laminar flow (Re<500):

(81) $$h_{conv,for}=3.83∙{V_{wind}}^{0.5}∙L^{-0.5}$$

for turbulent flow (Re>2000):

(82) $$h_{conv,for}=5.74∙{V_{wind}}^{0.8}∙L^{-0.2}$$

for mixed flow (500<Re<2000):

(83) $$h_{conv,for}=\left(5.74∙{V_{wind}}^{0.8}∙L^{-0.2}\right)-\left(16.46∙L^{-1}\right)$$

With,

(84) $$Re=\frac{V_{wind}\times L}{\nu }$$

Still, in order to define which characteristic length to define, it is important to evaluate the incidence angle of wind on the panel surface:

If $\left|\gamma_{PV}-\gamma_{wind}\right|<45{}^{\circ}$ then L is the module's height. If not, L is the module's width

Where $\gamma_{PV}$ is the PV's azimuth angle, while $\gamma_{wind}$ is the wind direction

• The third approach necessitates the evaluation of heat transfer coefficient as follows:

(85) $$h_{conv,for}=4.96\times {V_{wind}}^{0.5}\times L^{-0.5}$$

The accounted mechanism of both forced and natural convection can be considered through the calculation of a Nusselt mean value, according to the following expression:

(86) $$\bar{{Nu}^{3}}=\bar{{Nu}_{nat}^{3}}+\bar{{Nu}_{for}^{3}}$$

(87) $$h_{conv}=\frac{\bar{Nu}∙\lambda_{air}}{L}$$

2. View Factor

The view factors can be defined as follows [45]:

(88) $$F_{fg-sky}=\frac{1}{2}\left(1+\cos\left(\theta \right)\right)$$

(89) $$F_{fg-gr}=\frac{1}{2}\left(1-\cos\left(\theta \right)\right)$$

(90) $$F_{Ted-sky}=\frac{1}{2}\left[1+\cos\left(\pi -\theta \right)\right]$$
